# The Continuing Debate on Deep Molluscan Phylogeny: Evidence for Serialia (Mollusca, Monoplacophora + Polyplacophora)

**DOI:** 10.1155/2013/407072

**Published:** 2013-11-21

**Authors:** I. Stöger, J. D. Sigwart, Y. Kano, T. Knebelsberger, B. A. Marshall, E. Schwabe, M. Schrödl

**Affiliations:** ^1^SNSB-Bavarian State Collection of Zoology, Münchhausenstraße 21, 81247 Munich, Germany; ^2^Faculty of Biology, Department II, Ludwig-Maximilians-Universität München, Großhaderner Straße 2-4, 82152 Planegg-Martinsried, Germany; ^3^Queen's University Belfast, School of Biological Sciences, Marine Laboratory, 12-13 The Strand, Portaferry BT22 1PF, UK; ^4^Department of Marine Ecosystems Dynamics, Atmosphere and Ocean Research Institute, University of Tokyo, 5-1-5 Kashiwanoha, Kashiwa, Chiba 277-8564, Japan; ^5^Senckenberg Research Institute, German Centre for Marine Biodiversity Research (DZMB), Südstrand 44, 26382 Wilhelmshaven, Germany; ^6^Museum of New Zealand Te Papa Tongarewa, P.O. Box 467, Wellington, New Zealand

## Abstract

Molluscs are a diverse animal phylum with a formidable fossil record. Although there is little doubt about the monophyly of the eight extant classes, relationships between these groups are controversial. We analysed a comprehensive multilocus molecular data set for molluscs, the first to include multiple species from all classes, including five monoplacophorans in both extant families. Our analyses of five markers resolve two major clades: the first includes gastropods and bivalves sister to Serialia (monoplacophorans and chitons), and the second comprises scaphopods sister to aplacophorans and cephalopods. Traditional groupings such as Testaria, Aculifera, and Conchifera are rejected by our data with significant Approximately Unbiased (AU) test values. A new molecular clock indicates that molluscs had a terminal Precambrian origin with rapid divergence of all eight extant classes in the Cambrian. The recovery of Serialia as a derived, Late Cambrian clade is potentially in line with the stratigraphic chronology of morphologically heterogeneous early mollusc fossils. Serialia is in conflict with traditional molluscan classifications and recent phylogenomic data. Yet our hypothesis, as others from molecular data, implies frequent molluscan shell and body transformations by heterochronic shifts in development and multiple convergent adaptations, leading to the variable shells and body plans in extant lineages.

## 1. Introduction

**Figure 1:**
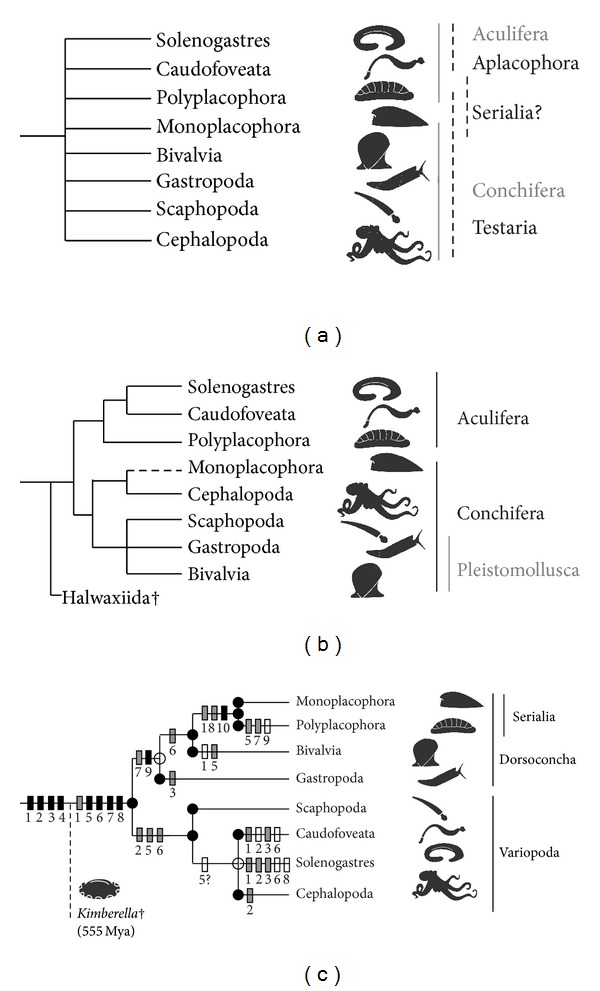
Schematic trees of molluscan relationships. (a) showing traditional proposed subdivisions. (b) consensus tree of two recent molluscan phylogenies inferred from large-scale genomic data by Kocot et al. [5] and Smith et al. [8]. The traditional concepts of Aculifera and Conchifera are supported but with differing positions of scaphopods. Monoplacophora is missing in the data set of Kocot et al. [5] (dotted line reflects the position of Monoplacophora in Smith et al. [8]). (c) the preferred multilocus tree with morphological features indicated numerically on branches. Unfilled dots indicate maximum Bayesian node support, filled dots additional high (>75%) bootstrap support in ML analyses. The Ediacaran fossil genus *Kimberella* corresponds to the description of molluscan stem-group features (1–4, below); crown group taxa originating in the Cambrian and later are united by additional features. Black boxes indicate first appearance of features; grey boxes indicate significant adaptive change; unfilled boxes indicate trait reversals: (1) radula: bipartite in stem molluscs and paedomorphic aplacophorans; broadened, on cartilages and specialised in crown molluscs, stereoglossate-like in Serialia; lost in Bivalvia (and several gastropods); (2), foot with broad gliding sole: transformed into digging foot in variopods (and derived bivalves), narrowed and reduced in aplacophorans, and forming the funnel in cephalopods; (3) circumpedal mantle cavity, miniaturised and anteriorly dislocated in torted gastropods while placed posteriorly in vermiform molluscs; (4) separate mantle covered with cuticula (with calcareous spicules in chitons, aplacophorans, and probably* Kimberella*); (5) dorsal shell: duplicated/fragmented in bivalves and chitons, lost in aplacophorans (and members of most other classes); (6) head with paired appendages: multiplied into feeding tentacles in variopods; trait for head reduction in bivalves plus Serialia and aplacophorans; (7) pericardium: heart fused around intestine in Dorsoconcha; (8) paired ctenidia: expanded to serially repeating gills in Serialia (and nautiloid cephalopods) and reduced in Solenogastres and some gastropod lineages; (9) complex stomach with style (reduced in carnivorous subgroups and chitons; convergently (?) present in a caudofoveate family); (10) paired eightfold dorsoventral muscles; (11) (not shown) statocysts (lost convergently in chitons and aplacophorans); (12) (not shown) suprarectal visceral commissure (subrectal convergently in chitons and aplacophorans).

Molluscs are a morphologically megadiverse group of animals with expansive body plan modifications. There is no doubt about the monophyly of Mollusca as a whole or of any of the eight extant molluscan classes, based on strong morphoanatomical evidence and the consensus of molecular studies [1]. Despite a number of important recent studies, resolving ingroup molluscan topology remains contentious (Figure 1(a)) and a major challenge of invertebrate evolution [2]. 

Other studies have not had access to suitable material for broad taxon sampling, in particular for monoplacophorans, a class of small deep-sea molluscs that still remain rare and largely inaccessible [3, 4]. Among several recent studies on molluscan phylogeny, most use a subset of classes [5–7]; only one phylogenomic study so far has included all eight classes [8]. 

Multigene studies on ribosomal proteins [6] and housekeeping genes [7] and two broad phylogenomic (EST-based) data sets [5, 8] supported a monophyletic clade Aculifera. This clade comprises those molluscs with a partial or entire body covered by a cuticle with calcareous spicules or scales and is composed of shell-less vermiform molluscs (aplacophoran) and shell-plate bearing Polyplacophora (chitons). The opposing clade Conchifera (incorporating the five classes with a “true” shell) remains controversial; phylogenomic studies recovered a monophyletic clade Conchifera [5, 8], but ribosomal protein multigene and housekeeping gene analyses showed paraphyletic Conchifera [6, 7]. 

A contradictory alternative hypothesis was proposed by earlier ribosomal RNA-dominated multilocus studies that included Monoplacophora and recovered this class as the sister to Polyplacophora [4, 9, 10]. This clade “Serialia” combines conchiferan and aculiferan members and is thus incompatible with results of recent molecular studies or the morphological Testaria (i.e., Conchifera + Polyplacophora) hypothesis (Figure 1). This result was widely criticised in the literature (e.g., [11]). Yet initial deficiencies [12] of the study by Giribet et al. [9] were addressed by Wilson et al. [10] and Serialia recovered again in a partially overlapping data set by Meyer et al. [13] and independently by Kano et al. [4]. 

The single phylogenomic data set with a monoplacophoran species also indicated some signal for Serialia, though weaker than that supporting a relationship of cephalopods and monoplacophorans within Conchifera [8]. Phylogenomic data sets cannot yet cover the same density of taxon sampling relative to targeted gene approaches, and while systematic errors of phylogenomic analyses have been explored recently (e.g., [14–16]), there is already a suite of tools available for addressing well-known pitfalls of ribosomal RNA-based sequences (e.g., [17–20]). All data sets may still contribute to ongoing investigations of phylogeny if used and interpreted with care.

Where published topologies differ radically from concepts born from morphoanatomical hypotheses, these results have often been dismissed as artefacts even by the studies' own authors. In addition to the “Serialia” concept, several studies over the last decade have repeatedly recovered Caudofoveata sister to Cephalopoda (e.g., [6, 9, 10, 21–23]). But this pattern has low support values [6, 12]. The position of scaphopods is also highly variable, sometimes in a clade with gastropods and bivalves [5, 7, 8] or sister to aplacophorans and cephalopods [9, 10, 21]. With only eight major clades to rearrange, it could be a serious handicap that many studies exploring molluscan topology have had to exclude one (e.g., [5, 7, 21]) to three (e.g., [4, 6]) classes, and all but one previous study [10] used single-taxon exemplars for at least one [9] to as many as three [7, 8] of those clades. More and better quality data from the monoplacophorans are necessary to resolve molluscan relationships and particularly the two mutually exclusive hypotheses Serialia and Aculifera. We assembled a large multilocus data set for molluscs, including novel sequences of three monoplacophoran species (added to previously published data for only two species, *Veleropilina seisuimaruae* and *Laevipilina hyalina*). To determine the plausibility of this new topology, we applied several tests for phylogenetic informativity, saturation of sites, and compositional heterogeneity within the molecular data sets and have also considered our results against other molecular, morphological, and fossil evidence. Finally we calculated a new time tree via a relaxed molecular clock approach, using multiple sets of fossil calibration points.

Applying carefully calibrated molecular clocks on broad extant taxon sets and reconstructing characters on dated ancient lineages are indispensable for interpretation of enigmatic key fossils such as *Halkieria* or *Nectocaris* that may form part of the early evolutionary history of the group (e.g., [24–27]). We present an alternative view on molluscan evolution that supports the Serialia hypothesis and demonstrates that the debate on pan-molluscan relationships is still in progress.

## 2. Material & Methods

### 2.1. DNA Extraction, PCR, and Sequencing

DNA from 12 molluscan taxa, including 3 previously unsampled monoplacophoran species, was extracted using the Qiagen Blood and Tissue Kit (Qiagen, Hilden) by following the manufacturer's instructions. Amplifications of the four standard marker fragments, partial 16S, partial 18S, partial 28S, and complete H3, were carried out under PCR conditions and with primer pairs shown (see Supplementary Material available online at http://dx.doi.org/10.1155/2013/407072). Sequencing reactions were operated on an ABI 3730 48 capillary sequencer of the sequencing service of the Department of Biology of the LMU Munich by using the amplification primers. Newly generated sequences were edited in Sequencher version 4.7 (Gene Codes Inc., Ann Arbor, MI, USA).

### 2.2. Taxon and Gene Sampling

To compile a comprehensive and dense taxon sampling for resolving deep molluscan relationships, we expanded earlier published data sets [9, 10] by our own and archived (Genbank) data, including a broad selection of outgroups and initially including any molluscs with substantial sequence information available for five standard marker fragments (partial 16S rRNA, partial or complete 18S rRNA, partial 28S rRNA, complete H3, and partial COI). In some poorly sampled but significant ingroup clades we also included species with fragmentary sequence data. Previously unpublished, partial 16S, complete 18S and 28S, complete H3, and partial COI sequences of *Veleropilina seisuimaruae *were provided separately by one of the authors (YK). The total initial data set comprised 158 taxa (141 molluscan and 17 outgroup taxa; Suppl. Table 2). 

### 2.3. Data Cleaning and Alignment

All the downloaded and new single sequences, including all 28S sequences, and all individual amplicons for 18S sequences in Solenogastres, were cross-checked against the nucleotide database of BLAST [29] by using the blastn algorithm. Potentially aberrant or problematic fragments were removed from the data sets (Suppl. Table 3A). 

In some bivalve 28S sequences a dubious part of ca. 500 bp was detected in an otherwise homogeneous molluscan alignment. This portion differed substantially in most bivalve taxa but not in all and was highly heterogeneous also in closely related species. No pattern could be observed, so we removed the dubious region (Suppl. Table 3B). 

The 18S sequences of Solenogastres were partially excluded due to contamination. Retained sequences of *Epimenia* species (*E. *sp.,* E. australis, *and* E. babai*) were aligned separately with the first uncontaminated sequences of Meyer et al. [13], and resulting large gaps were cut by hand according to the template sequences of *Micromenia fodiens, Simrothiella margaritacea *and* Wirenia argentea* (Meyer sequences in [13]).

Patellogastropoda has aberrant 18S and 28S sequences with many indels causing highly incongruent alignments (own observations), leading to long branches and attraction artefacts in previous [13] and our own analyses. Patellogastropoda clustered with long branched Cephalopoda and Solenogastres under different regimes (Table 1). To verify the correct position of Patellogastropoda within or outside other Gastropoda a more focused data set was generated comprising only gastropod taxa plus some selected, short-branched outgroup taxa, that is, two bivalves, two polyplacophorans, one annelid, and one kamptozoan. This alignment is more homogeneous, and patellogastropods appear as a moderately long branch in a rather derived position within the Gastropoda (Suppl. Figure 2). So we confirm that patellogastropods show aberrant evolution leading to long branch attraction artefacts in broader data sets [13]; therefore we excluded this clade from the main analyses.

Single alignments (per fragment) were created with Mafft version 6.847b [30] with the implemented E-INS-i algorithm. Alignments of 16S, 18S, and 28S rRNA were masked with Aliscore version 5.1 [17, 31] by running 10,000,000,000 replicates. All ambiguous positions were automatically cut with Alicut version 2.0 [17, 31] to remove highly variable positions that could lead to aberrant phylogenetic signals. The alignments of protein coding genes H3 and COI were manually checked for stop codons using MEGA5 [32]. The single data sets were concatenated automatically using FASconCAT version 1.0 [33]. This procedure resulted in a total alignment of 142 taxa with 8721 bp in length and a proportion of 60% gaps (Suppl. Table 5). Where taxon sampling had to be modified, for example, removing taxa or dubious gene fragments, this was done in the initial single data sets and the complete procedure of alignment, masking and concatenation was carried out again.

Final analyses were computed with the large data set excluding Patellogastropoda (142-taxon set), a targeted taxon subset (81-taxon set, alignment length 8367 bp, proportion of gaps 57%) after pruning fast-evolving species or derived members of densely sampled undisputed clades, and the gastropod data set (all gastropods including Patellogastropoda plus selected slowly evolving outgroups). Moreover, we generated and analysed diverse data sets for control reasons to test interclass topologies: the 142- and 81-taxon sets without Aplacophora, the 142-taxon set without long-branched Cephalopoda and Solenogastres, the 142-taxon set with COI and H3 coded as amino acids (142-taxon set amino acid), and one data set that comprises only 18S, 28S, and H3 fragments of the 142-taxon set (Suppl. Table 5). The concatenated sequence matrices of the two main analyses (142-taxon set and 81-taxon set) were deposited at TreeBase (http://purl.org/phylo/treebase/phylows/study/TB2:S14594). New sequences generated herein were deposited at Genbank (Suppl. Table 2). 

### 2.4. Preanalyses of the Data

Since saturated sequences have minimal or no phylogenetic signal and could even lead to anomalous results, we measured substitution saturation of the protein coding genes, namely, H3 and COI, with Xia's method implemented in DAMBE version 5.2.31 [37]. We used default parameters, and the proportion of invariable sites was specified. The method was executed for all three codon positions together, for combined first and second codon positions, and for third codon position separately. In both cases, H3 and COI, the index of substitution saturation (Iss) values of all three codon positions in combination were significantly smaller than critical index of substitution saturation (Iss.c) values. This was also true for the alignments of first and second codon positions. This assumes that those positions conserve phylogenetic signal and are useful for further analyses. In the case of third codon positions only, substantial saturation could be observed (Iss significantly higher than Iss.c). All results are shown in Supplementary Table 6. Although substitution saturation was observed in third codon positions of H3 and COI, we ran additional analyses with the complete sequence information (1st, 2nd, and 3rd codon positions) to implement potential phylogenetic signal for lower taxonomic levels.

To crosscheck the phylogenetic results of the data sets with and without excluded third codon positions of protein coding genes we conducted the same analyses with all three codon positions included, using distinct models of evolution for the three different codon positions and without third codon positions of H3 and COI. 

Testing the evolutionary models for all genes and in case of COI and H3 for every single codon position and for codon positions one and two versus position three was carried out with the programs Modeltest version 3.7 [38] (for complete alignments) and MrModeltest version 2.3 [39] (for codon positions) by the help of PAUP* version 4b10 for Windows [40]. With the amino acid alignments of H3 and COI we additionally tested for the best fitting amino acid model of evolution using ProtTest version 2.4 [41]. As RAxML provides only a part of the models that can potentially be tested by ProtTest we only selected those models in our ProtTest analysis (DAYHOFF, DCMUT, JTT, MTREV, WAG, RTREV, CPREV, VT, BLOSUM62, and MTMAM). The resulting best models for all genes (16S, 18S, 28S, H3, and COI), distinct codon positions of H3 and COI, and amino acid alignments of H3 and COI as well as the corresponding proportions of invariant sites and the gamma distribution shape parameters are shown in Supplementary Table 4. 

### 2.5. Phylogenetic Analyses

Maximum Likelihood (ML) analyses for all data sets were executed using RAxML-HPC for Windows [28] and RAxML version 7.2.6 [28] on the Linux cluster of the Leibniz Computer Centre. Parameters for the initial rearrangement settings and the rate categories were optimised under the GTRCAT model of evolution and a partition by genes (16S, 18S, and 28S) and codon positions (COI, H3) by conducting the hardway analysis described by Stamatakis [42]. 

First, a set of 10 randomised Maximum Parsimony (MP) starting trees was generated. Second, based on this set of starting trees, the ML trees with a specified setting of initial rearrangements (−i 10) and with an automatically determined initial rearrangement setting had to be inferred. Third, the number of rate categories was adjusted. Initial setting –c 10 was augmented by increments of 10 up to –c 50 for all MP starting trees. The fourth step was to execute 200 inferences on the original alignments. Finally, values of 1000 bootstrap topologies were mapped on the best-scoring ML tree.

Bayesian analyses for selected data sets were conducted with MrBayes v. 3.1.2 [43]. Partitioning with corresponding models of evolution, substitution rates and nucleotide frequencies were applied according to the results of Modeltest [38], MrModeltest [39], and ProtTest [41]. One tree was sampled every 1000 generations. If the average standard deviation of split frequencies declined 0.01 after 5 million generations the analysis was stopped. If not, analysis was continued with another 5 million generations. If the average standard deviation of split frequencies still did not decrease, the log likelihood values were examined with Tracer version 1.5 [44]. If the run reached stationarity, the analysis was stopped. Burn-in was set to 2500 after 5 million generations and to 5000 after 10 million generations.

### 2.6. Molecular Clock Analyses

Time estimations were performed with the software package BEAST version 1.6.1 [34]. The program is based on the Bayesian Markov Chain Monte Carlo (MCMC) method and therefore can take into account prior knowledge of the data. That is used when nodes in the topology are calibrated and the rate of molecular evolution along the branches is estimated. 

We used nine fossil calibration points (Suppl. Table 7) with their corresponding prior distributions and assumed a relaxed clock with a lognormal distribution [45] of the rates for each branch (Suppl. Table 7). This setting is recommended because it additionally gives an indication of how clock-like the data are [46]. Calibration points were set with a minimum bound according to Jörger et al. [47]. To reduce computing time we used the targeted (81-taxa) data set for time estimations. The topology was constrained according to the resulting tree of the phylogenetic analyses. 

An Xml-file with all information on data, calibration points, priors and the settings for the MCMC options was created with BEAUti version 1.4.7 [34]. Gamma-shaped priors for all nine calibration points were used (Suppl. Table 7). We assumed that the lower bound of each calibration point is not more than 10% of its maximum age. In case that the next older fossil is within these 10% boundary we used the maximum age of that fossil as lower bound for the younger fossil [48].

Detailed partitioning of genes (16S, 18S, and 28S) and codon positions of COI and H3 and the constraint tree topology were added by hand to the Xml-file. The analysis was executed for 30 million generations, sampling one tree every 1000 generations on the Linux cluster of the Leibniz Computer Centre. The implemented program Tracer version 1.5 [44] was used to confirm that posterior probabilities had reached stationarity. Burn-in was set to 25% (7500), so 22,500 trees were effectively analysed with TreeAnnotator version 1.6.1 [34] to form the summary tree. Further, to check the reliability of our fossils, we repeated the same analysis several times and always omitted one calibration point (Table 2; Suppl. Table 7). 

### 2.7. Testing Hypotheses

Several existing hypotheses about the molluscan interrelationships (Table 3) were tested by executing Approximately Unbiased tests (AU tests) implemented in Treefinder version of October 2008 [36]. Therefore the input constraint trees were computed with RAxML-HPC [28] by using the –g-option and the associated partition by genes and codon positions. Those input tree topologies were tested in Treefinder with maximum number of replicates under the GTR model. 

## 3. Results and Discussion

### 3.1. Analyses

Analysing traditional multilocus markers for several large taxon sets with Maximum Likelihood and Bayesian methods under different alignment and masking regimes (Table 1, Suppl. Table 5), we recovered consistent phylogenetic trees (Figure 1(c)) with monophyletic Mollusca in contrast to other studies with similar markers [9, 10, 19, 21] and strong support for the monophyly of all molluscan classes, including Bivalvia (also in contrast to some earlier studies [9, 19, 21]). 

Our approach included rigorously testing of all amplicons before and after alignment, which led to the exclusion of aberrant or problematic, previously published sequences from the data set (Suppl. Table 3). Criticism of previous accounts using the same set of markers has included the incomplete representation of taxa and the varying extant of missing data [12, 49]. Missing data is a common burden of multilocus studies and will be more severe for phylogenomic approaches [14, 15]. Our preanalyses showed that dubious sequences or ambiguous parts of alignments had much greater effect on the outcome than selecting taxa with the highest amount of data available. Rather than maximizing sequences per species, we concentrated on increasing taxon sampling to minimise potential branch lengths. Our quality controlled 158-taxon set includes 17 lophotrochozoan outgroups. Analytical trials on different subsets of nonmolluscan outgroups altered outgroup topology and support values of some basal ingroup nodes but did not change the ingroup topology (Figure 1). 

**Figure 2:**
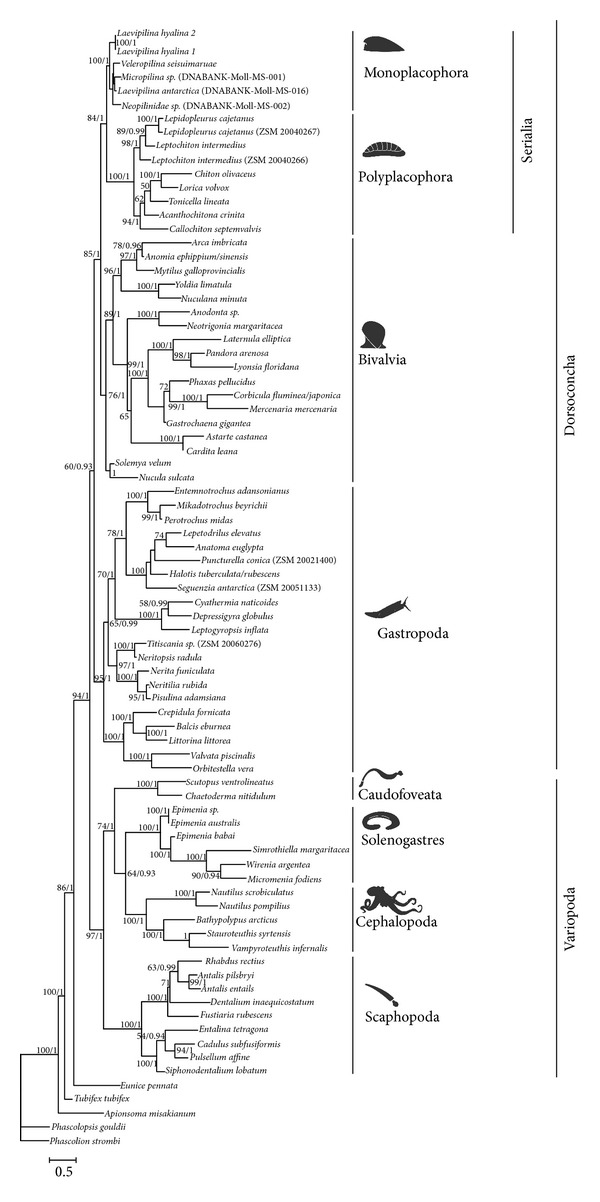
Preferred molluscan tree. Maximum Likelihood analysis (RAxML [28], hardway) of pruned 81-taxon set; values at nodes refer to bootstrap support (1000 pseudoreplicates, first value) and posterior probabilities obtained from the Bayesian analysis (second value).

Alignment issues involved in ribosomal RNA data were addressed by an array of measures proven to be beneficial ([20]; see Section 2). Potential homoplasy in protein coding genes (especially the third codon positions) in our preferred multilocus analysis was addressed by additionally running the analysis with those fragments (COI and H3) encoded as amino acids. This had little effect on the topology but supported monophyletic Aplacophora. We applied a variety of alignment tools, including masking (Aliscore [17]) and refinement algorithms based on secondary structures (RNAsalsa [18]) and applied compartmentalised analyses of taxon clusters causing obvious alignment problems. Excluding patellogastropods (142-taxon set, Suppl. Figure 1; see Section 2) did not change our molluscan backbone topology (Figure 1(c)) but improved alignments. Separately analysing gastropods plus some slowly evolving outgroup taxa shows patellogastropods cluster with vetigastropods (Suppl. Figure 2). Our main aim was to elucidate molluscan relationships at the class level; thus we further pruned outgroups and fast-evolving members from more densely sampled ingroups (such as heterobranch gastropods) and used an 81-taxon set presented here in our main analysis (Figure 2). 

### 3.2. The Basal Molluscan Dichotomy

In our new tree, the phylum Mollusca is divided into two clades (Figure 1(c), Figure 2, Suppl. Figure 1). The first clade is composed of Gastropoda sister to a clade of Bivalvia and Serialia (Monoplacophora + Polyplacophora). For convenience we will refer to this clade as “Dorsoconcha”; the name refers to the (plesiomorphic) presence of a dorsal shell for members of this clade, though modified to two lateral valves in bivalves and to (7-)8 dorsal plates in chitons, and the shell internalised or lost multiple times especially among gastropods. 

Gastropods, bivalves, and monoplacophorans are commonly considered to be united by their single shell (secondarily split in bivalves) built by a shell gland at the mantle border (and by the entire mantle roof secreting organic matrix and calcareous layers letting the shell grow thicker, or repair damage). Chitons are traditionally excluded from the hypothetical clade “Conchifera” on the basis of their eight shell plates. The chiton girdle is also covered by a cuticle with embedded calcareous and organic sclerites, similar to the body cuticle of the shell-less aplacophorans, but according to our results, this is convergent and may reflect the different, single versus multicellular spicule formation in these taxa [50]. That chitons cluster with monoplacophorans rather than aplacophorans is congruent to previous molecular approaches that included monoplacophoran exemplars [4, 9, 10, 13]. The exception is the phylogenomic study by Smith et al. [8], in which a single monoplacophoran, *Laevipilina hyalina*, robustly clustered with cephalopods in the main analyses, though parts of the genes used also showed signal supporting an association with chitons. 

In the second major molluscan clade, Scaphopoda are sister to a clade of vermiform Caudofoveata and Solenogastres, plus Cephalopoda. Herein we will call this clade “Variopoda,” referring to the various derived foot attributes of its members: the digging foot in Scaphopoda, reduced narrow gliding sole or completely lost in (adult) aplacophorans, and transformed in cephalopods possibly building parts of tentacles and funnel. Dorsoconcha appears as a monophyletic group although bootstrap support is low (60%), and the Variopoda is strongly supported in all Maximum Likelihood analyses; Bayesian posterior probabilities are high for both nodes (Figure 2, Suppl. Figure 1). 

The placement of aplacophorans within Variopoda is unconventional, but a sister relationship between Scaphopoda and Cephalopoda has been previously put forward [51, 52]. Previous multilocus approaches with broad taxon sampling (i.e., more than one exemplar of each aplacophoran class) are actually not in general disagreement with Variopoda, since contaminated aplacophoran sequences may account for occasionally aberrant topologies [9, 10, 13]. Inner scaphopod topology resolves the two currently recognised groups Dentaliida and Gadilida, as does Cephalopoda splitting into modern Nautilida and Coleoidea, and is congruent with previous classifications [53].

**Figure 3:**
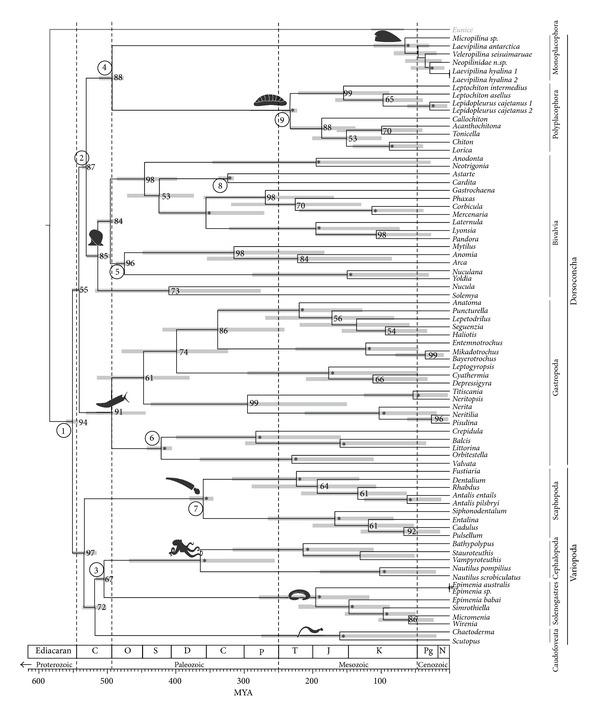
Chronogram of molluscan evolution. Divergence times (million years before present, Ma) estimated from BEAST version 1.6.1 [34] under an uncorrelated lognormal relaxed clock model; bars refer to the 95% highest posterior density. All nodes show maximum posterior probabilities (1.0, not indicated) from a run with 10^8^ generations (25% burn-in). Numbers at nodes refer to bootstrap support values (>50%; asterisks are 100%) obtained from separate Maximum Likelihood analysis (RAxML [28], hardway, 1000 pseudoreplicates) of the same data set. Circled digits indicate calibrated nodes. Details of calibration can be found in Supplementary Table  7. Omitting Cambrian calibrations shifts molluscan diversification deeper into the Precambrian (for sensitivity analyses see Table 2).

We calculated time trees with a Bayesian molecular clock approach (Figure 3) using a mix of younger and older calibration points (Suppl. Table 7). We also tested sets of calibrations successively excluding each single calibration point used (Table 2) to minimise circularity involved by calculating individual node times [54]. All our time trees confirm a Precambrian origin of Mollusca (Table 2, Suppl. Table 8) in agreement with previous studies [7], and 95% confidence time bars of all our time trees allow for a Cambrian origin of those classes with a reliable fossil record (Figure 3). As a further sensitivity test we also calculated a time tree from a data set excluding aplacophorans; the topologies are congruent and node ages almost identical, confirming general time estimates (not shown).

Molluscan diversification occurred at an extremely rapid pace after the initial origination of the shell (Figure 3). Short branches at the base of the ingroup can be artefacts of signal erosion in deep nodes [55], but as we discuss below, the rapid early evolution of Mollusca is also supported by the fossil record. Our molecular clock indicates a potential time frame of only around 20–40 million years from the first shelled molluscs (ca. 560–540 Ma) to the presence of differentiated variopod, dorsoconch, gastropod, bivalve, and serialian stem lineages (ca. 520 Ma). The shell was central for rapid evolutionary success of molluscs, and shell modification and divergence are correlated with adaptive radiations during this early period.

### 3.3. Evaluating Molecular Data Sets

All recent multigene and phylogenomic studies [5–8] have tested the effects of gene sampling, analytical methods, and inference programs; like our results, their topologies were more or less robust, also against varying outgroup selection. Sensitivity analyses do not attribute the major split into Variopoda (or parts thereof) and Dorsoconcha or the recovery of Serialia to LBA effects. Yet our multilocus study uses fewer markers and nucleotides than “next-generation sequencing” studies [5–8], so it may be more prone to inadequate signal of certain markers or stochastic errors. 

Split decomposition analyses of an earlier multilocus set [9] usually recovered the single monoplacophoran species among bivalves [12], consistent with a Dorsoconcha clade. Splitstree analyses (not shown) of our improved data set still show overall polytomy and some individual taxa are clearly misplaced in the network (e.g., the gastropod *Crepidula* clusters with cephalopods). Overall, most dorsoconch terminals are separated from variopods. Within Dorsoconcha, monoplacophorans cluster with chitons and bivalves. A lack of tree-like structure and *a priori* split support, especially in a large and heterogeneous taxon set, may not necessarily mean that there is too little signal for phylogenetic analyses; it just means that there is conflict that may or may not be resolved applying current models of sequence evolution. 

Nuclear ribosomal RNA genes were shown to be informative even on deeper levels than basal molluscs, if treated adequately [20]. Other, supposedly faster-evolving mitochondrial markers (partial COI, 16S) were stringently masked herein, partitioned when necessary or excluded when saturated (Suppl. Tables 4–6). Combined analysis incorporates multiple tempos of evolution experienced by the different loci and is therefore more representative of deep evolutionary patterns. Our backbone topology is robust against varying the taxon and marker sets, masking and partitioning regimes, models of evolution, and methods of analyses (Table 1, Suppl. Tables 4 and 5). 

### 3.4. Evaluating Alternative Morphological and Molecular Concepts

We directly evaluated the statistical fit of major competing morphology- or molecular-based concepts constraining our topologies and calculating their likeliness according to our data set. Using our preferred 81-taxon set with all markers, but also under most other schemes, the AU test rejects all the higher molluscan textbook concepts [1]: the Testaria, Aculifera, Conchifera, Cyrtosoma, and Diasoma hypotheses, with the highest possible statistical support; the same AU tests do not reject Dorsoconcha nor Variopoda (Table 3). We also tested our data against three new molecular concepts (Figure 1(b)): Pleistomollusca (Bivalvia + Gastropoda) established by Kocot et al. [5] and the clades of Monoplacophora and Cephalopoda [8] versus other conchiferans (Scaphopoda, Gastropoda, and Bivalvia) [5, 8]. Only the clade of Monoplacophora and Cephalopoda was not rejected with significant support in any of the main analyses, but all these groups received much lower AU values than our unconstrained topology. 

While several recent phylogenomic studies recover Aculifera [5, 7, 8], the Serialia concept has been tested only by Smith et al. [8], by inclusion of a single monoplacophoran species. Though association with cephalopods is preferred, there is a weaker signal also for Serialia [8]. Kano et al. [4] recovered Serialia but did not include any aplacophoran taxa in their data set. The Serialia as a concept cannot be dismissed yet, and our dense taxon sampling herein, though based on far fewer sequences than recent phylogenomic approaches [5, 8], still may allow for a more differentiated and perhaps more correct view on molluscan interclass relationships. 

The association of cephalopods and aplacophorans has been recovered previously but dismissed as an artefact of high substitution rates in rRNA genes [6, 13, 21]. But our results cannot easily be explained by long branch attraction (LBA) effects (*contra* [13]). Branch lengths of scaphopods and caudofoveates are moderate, and the variopod node is stable against removal of putative long branched taxa showing accelerated evolutionary rates or biased base compositions [13], such as the branches of Solenogastres or Cephalopoda or both (trees not shown). 

Molluscan evolution, whatever the underlying tree, is known to be laden with convergence at all taxon levels, including morphological features previously suspected to be informative for deep phylogeny (e.g., [56–58]). Conclusions derived from single organ systems, or the shell alone, are not able to exclude alternative interpretations. Coding hypothetical bauplans rather than existing representatives has been criticised [59, 60] and may lead to erroneous assumptions especially in groups with uncertain internal topology such as gastropods or aplacophorans. Morphocladistic approaches to date (e.g., [61–63]) all recovered Testaria, but this hypothesis is not supported by any molecular approaches. 

Our proposed topology and any other nontestarian hypothesis imply that ancestral molluscs were complex rather than simple. This means that many anatomical characters inherited by descendants may be plesiomorphic and thus not informative, or could have been reduced or lost repeatedly, implying a high level of homoplasy. In fact, early molluscan phylogeny may have been shaped by habitat-induced selective pressure combined with heterochronic processes (e.g., [64]). This combination may lead to concerted morphological parallelisms powerful enough to obfuscate any phylogenetic signal, which has been found to be the case in heterobranch gastropods (e.g., [47, 65, 66]). It is possible to disentangle even highly homoplastic and heterochronic groups (e.g., [67–69]) if detailed and reliable microanatomical data are available on a dense ingroup taxon sampling, which is, however, not yet available for most molluscs. Unfortunately none of the many competing morphology-based hypotheses on molluscan class interrelationships available at present appears to represent a reliable benchmark for evaluating molecular topologies. 

### 3.5. Topologies Tested against the Fossil Record

Molluscan diversification has been widely assumed to originate from a basal “monoplacophoran” bauplan [59], although early single shelled molluscs cannot be reliable separated from gastropods or any nonmonoplacophoran univalve [70]. The earliest calcareous molluscan-like shells, including undisputed molluscs, appear in the uppermost Precambrian, in the late Nemakit-Daldynian ca. 543 Ma [70]. Polyplacophoran shell plates first appear in the Late Cambrian, almost 50 My later [7, 71]. This does not support the Testaria hypothesis that would suggest that chitons evolved before the invention of a true “conchiferan” shell. There are dubious disarticulated microscopic chiton-like plates [72] from the early Meishuchunian (likely Early Tommotian) of China, but these still appeared later rather than earlier than the very first undisputed conchiferan shells. The Aculifera concept with monoplacophorans sister to other members of Conchifera or our molecular basal dichotomy are both fully compatible with the origin of molluscan shells latest at the Precambrian/Cambrian boundary. 

The earliest tryblidian monoplacophorans are recorded from the Late Cambrian [73]. Older, nontryblidian “monoplacophorans” do not show serialised muscle scars and thus cannot be considered part of the crown-group. Yet the earliest reliable bivalves with elaborated hinge and ligament (*Fordilla*, *Pojetaia*) appear earlier, in the Early Tommotian ([74]; ca. 535 Ma). Both Aculifera and our basal dichotomy are not contradicted by the early appearance of bivalves. Under an Aculifera topology, chiton-like stem members could appear soon after a terminal Precambrian split separating Aculifera and Conchifera. Interpreting Early to Middle Cambrian sachitids (halwaxiids) as stem aculiferans would help fill this gap [7], but these taxa show a chronological sequence of shell plate loss rather than acquisition, which may be contrary to a progressive transition to chitons. The mosaic taxon *Phthipodochiton*, which has been proposed as a stem aplacophoran, does not appear until the Ordovician [75, 76]; other fossils from the Silurian, combining aplacophoran and polyplacophoran features with some soft tissue preservation, have also been used to support the Aculifera hypothesis [77]. These could also simply represent further disparity in extinct Polyplacophora. Regardless, there is compelling evidence from molecular systematics as well as fossil evidence that aplacophorans lost their ancestral shell (or shell plates) secondarily, and many other groups show repeated shell-loss or evolution to a vermiform body plan. 

The topologies recovered by Vinther et al. [7] and Kocot et al. [5] support Aculifera but also imply that cephalopods are sister to Aculifera [7] or represent the earliest-diverging conchiferans [5] (excluding monoplacophorans from the analysis). However, there is no evidence for cephalopod-like fossils appearing earlier than, for example, bivalves. Similarly, bivalves are derived within Conchifera in the topology of Smith et al. [8], which is contradicted by the early fossil record of bivalves. In contrast, our basal dichotomy could fit with the many univalve small shelly fossils occurring earlier in the fossil record than bivalves, and both monoplacophorans and polyplacophorans appear later, actually at a similar time in the Latest Cambrian, and as predicted by a split of Serialia into Monoplacophora and Polyplacophora. 

### 3.6. The Timing of Early Molluscan Evolution

The molluscan stem is Precambrian according to all our molecular time trees. The Vendian (555 Ma) body fossil *Kimberella* was discussed as a mollusc [78], but not widely accepted as such, and rather treated as lophotrochozoan stem member or “no more specifically than as a bilaterian” [79]. According to previous constrained (e.g., [7]) and our less constrained time trees (Table 2, Suppl. Table 8), however, *Kimberella* appears late enough in the fossil record to be considered as a potential stem mollusc. The other recent molecular clock for Mollusca puts the stem Mollusca even deeper [4], but *Kimberella* is within the 95% HPD interval for the split of the basal dichotomy also recovered herein. Having confirmed the conceptual basis of our proposed topology is not rejected by evidence in the fossil record, we further consider the timing of the radiation of specific clades proposed by our molecular clock analyses (Figure 3). 

Cap-shaped Helcionellidae from the terminal Precambrian (e.g., *Latouchella*) are putative monoplacophorans according to the seminal study by Runnegar and Pojeta [80] or a separate molluscan class [81] or, based on nonserial muscle scars, gastropods [70]. Our time tree suggests that Nemakit-Daldynian and Earliest Tommotian molluscs with symmetrical cap-shaped shells with large openings are stem molluscs (or in the stem of one part of the basal dichotomy). In contrast, helicoid shells from the same period such as Aldanellidae (e.g., [82]) could well be gastropods, whether or not the animal was torted [70, 82].

Early Tommotian *Watsonella*, formerly known as *Heraultipegma* (the putatively earliest rostroconch), is a laterally compressed, bivalve-like univalve [70], possibly with dorsomedially decalcified or even bivalved shell [83]. This and other laterally compressed Watsonellidae may pre-date the first reliable Bivalvia (Early to Middle Tommotian *Fordilla*; [74] versus [70]) by some million years and thus could well be stem bivalves (or offshoots of the dorsoconch stem) according to our time tree (Figure 3). 

It is important to note that neither reliable Monoplacophora (*sensu* Tryblidia) nor reliable Polyplacophora (i.e., Paleoloricata) are known before Late Cambrian, and this is confirmed in our chronograms (Figure 3). Yu [84] interpreted the Early Cambrian Merismoconchia as having eight pairs of muscles on a pseudometameric shell, linking 8-plated chitons with single shelled monoplacophorans in a transitional row of shell fusion. The similarity of merismoconchs with both serialian classes is curious, and their early occurrence in the pretrilobite Meishucun Stage suggests they could be early stem Serialia. The microscopic merismoconchs with their ventrally still connected shell segments and seven observed pairs of muscle scars may have been a transitional stage in how to make a foot efficient for sucking and a shell more flexible to adapt to uneven hard substrates. According to our time tree (Figure 3), chiton-like shell “fragmentation” into fully separated plates occurred much later, after splitting from single-shelled monoplacophoran-like ancestors. 

The Cambrian (Atdabanian) *Halkieria* and related Middle Cambrian halwaxiids could also be interpreted as stem Serialia (Figure 3). A role as ancestral lophotrochozoans for halwaxiids as suggested by Edgecombe et al. [79] is not supported by our analysis.

According to our time tree (Figure 3), Yochelcionellidae, conspicuous Tommotian to Middle Cambrian shells that have a “snorkel,” could be part of the gastropod radiation as suggested by Parkhaev [70], or members of the dorsoconch stem lineage, or variopod stem members. The latter possibility is especially intriguing, since Yochelcionellidae evolved a “flow-through” water system with two shell openings; a dorsal shell elongates laterally and fuses ventrally, and the body axis shifts towards anterior growth extending head and foot out of a now tube-like shell. This condition is displayed by living and fossil variopods (i.e., scaphopods, cephalopods, and nonwatsonellid Rostroconchia).

Our results show that scaphopods could have split off from the variopod stem earlier, that is, in the Early Cambrian, but the oldest potential scaphopods in the familiar modern tusk-like shape are from the Ordovician [85] or even post-Devonian [86]. There is a vast record of Middle Cambrian tube-like shells that may be unrecognised parts of the early scaphopod diversification that started much earlier and morphologically less constrained than previously expected [87]. 

*Knightoconus*, a Middle to Late Cambrian large “monoplacophoran” conical shell with internal septa but no siphuncle [88], was described as a stem cephalopod [80] but subsequently questioned (e.g., [89]) and ultimately suspected to be a brachiopod [90]. *Knightoconus* could fit stratigraphically with stem cephalopods based on our evidence (Figure 3), but its morphological interpretation remains in doubt. The earliest reliable cephalopod fossils are the small bodied, septate, and siphuncle-bearing *Plectronoceras* from the Late Cambrian. Some versions of our analysis used *Plectronoceras* as a soft bound calibration point; by not using *Plectronoceras*, the origin of cephalopods shifts considerably towards the Silurian (Table 2).

Recently, shell-less and coleoid-shaped Lower Cambrian *Nectocaris pteryx* was regarded as a cephalopod [24], but this was immediately rejected on several lines of argument [91, 92]. Other putative Early Cambrian nectocaridids such as *Vetustovermis* [93] are superficially similar to *Nectocaris* in having a pair of long cephalic tentacles and stalked eyes but show a ventral foot separated from the supposedly wing-like mantle. Interpreting *Nectocaris* as having an axial cavity with gills and a funnel would provide synapomorphies for interpreting Nectocarididae as stem cephalopods [24, 94]. Molecular clock estimates can provide further insight to such contentious interpretations; according to our time estimates (which excluded nectocaridids as potential calibration points), *Nectocaris* is too ancient to be a cephalopod (Figure 3). If *Nectocaris *could be accepted as molluscan based on its contentious morphological interpretation, our time trees would be compatible with the idea that nectocaridids are stem variopods or within the stem of an aplacophoran/cephalopod or aplacophoran clade. Nectocaridid features with superficial similarities to coleoid cephalopods [24, 94] instead could be ancestral attributes of variopods: an anteriorly elongated body with head, long and flexible head tentacles, putative preoral hood, and a more or less reduced foot. 

The fossil record offers shells and body fossils which, by their occurrence and morphology, at least hypothetically fill our time tree with life. The topology and timing of our hypothesis of early molluscan evolution is not rejected by fossil evidence. 

### 3.7. Dorsoconcha

Molecular, morphological, and palaeontological evidence support (or fail to reject) our basal molluscan dichotomy. The clade Dorsoconcha includes most shelled molluscs and 98% of living species in four classes: Gastropoda, Bivalvia, Polyplacophora, and Monoplacophora. 

We note two inferred potential morphological synapomorphies of Dorsoconcha, both relating to the digestive system and both somewhat ambiguous: the intestine is surrounded by the pericardium in basal lineages of gastropods, bivalves, and in monoplacophorans and may be positionally homologous in chitons (Figure 1(c) character 7) and a rotating enzymatic crystalline style (or protostyle; Figure 1(c) character 9). Many basal, noncarnivorous molluscs have a more or less well-developed stomach separated into sorting zones, but only dorsoconchs (and a family of caudofoveates [61]) have the complex style; this was secondarily lost in chitons, which have a derived position in our proposed topology.

Most previous studies on the phylogeny of molluscs have been driven by the Conchifera concept [1, 95] and emphasised the opinion that Serialia violates putative conchiferan synapomorphies [12]. Such features all are plesiomorphic for dorsoconchs in our topology (Figure 1(c)). We note several potential apomorphies for Serialia (Figure 1(c)): the serial (seven or) eightfold (octoserial) dorsoventral pairs of muscle bundles, with two pairs of intertwined muscle bundles in chitons and also partly present in large *Neopilina* [95, 96]; serial gills in a circumpedal mantle cavity; a highly similar cerebral nerve cord; and a longitudinal elongation of the dorsoventrally flattened body, to mention just some (Figure 1(c)). The most prominent feature of Serialia, serial paired foot retractors, is also present in bivalves, but octoserial retractors appear in Ordovician *Babinka* and not in the earliest known bivalves in the Cambrian [97] (Figure 1(c) character 10). While head and buccal apparatus are reduced almost completely in bivalves, Serialia elaborated the buccal mass evolving highly similar radulae and the radula bolster. Similar foot and radula structures in patellogastropod limpets [61] could be either plesiomorphic or convergent, because Patellogastropoda are either an isolated early-diverging gastropod group or relatively recently derived within Vetigastropoda [98, 99]. 

From this topological result and the available fossil evidence, we propose that the last common ancestor of monoplacophorans and chitons was cap-shelled and adapted to epibenthic life in shallow waters, rasping algae or other microorganisms from rocky substrates (Figure 1). In this scenario, chitons are not primitive molluscs but rather a derived group, potentially adapted to high-energy marine shores. Monoplacophorans initially also were shallow water dwellers [73] but could have colonised deeper waters during the Palaeozoic, where modern monoplacophorans still occur [100]. The Cenozoic or Late Cretaceous molecular dating of the diversification of living monoplacophorans and their short inner branches ([4], Figure 3) are compatible with earlier assumptions of pronounced anagenetic changes in the long stem line of these so-called “living fossils” [4, 100]. 

### 3.8. Variopoda

The clade Variopoda (Figure 1(c)) groups the scaphopods, aplacophorans, and cephalopods together in all our analyses, and it is very well supported. We infer several features of variopods, including an apparent propensity for habitat-induced transformations (noted in the taxon epithet; Figure 1(c) character 2). Some other roughly hypothesised apomorphies may refer to a clade of scaphopods and cephalopods only, that is, to variopods only under the assumption that aplacophorans represent highly paedomorphic and thus aberrant offshoots (see below): lateral extension of a primitively dorsal cap-like shell forming a tube; twisting the growth axis during ontogeny from initial dorsoventral to an anterior body extension, translocating head foot and mantle cavity with anal opening anteriorly; formation of a ring-like dorsoventral muscle insertion; multiplication of cephalic tentacles into prey-capturing feeding tentacles; and at least partly using muscle antagonist rather than merely hydrostatic systems in these tentacles (convergently in gastropod cephalic sensory tentacles); a hood is formed anterior to the mouth; and muscular retraction of the foot is used to pump water, waste, and gametes through/out the mantle cavity. 

A clade of scaphopods and cephalopods repeatedly has been proposed based on morphological data, sometimes with one or the other or both together allied with gastropods [1], and was recovered by molecular data [52] and broadly within some pan-molluscan molecular phylogenies [10, 21]. In contrast, morphocladistic neontological [101] and palaeontological studies (e.g., [80, 102]) advocated the Diasoma concept suggesting scaphopods as sister to bivalves with a rostroconch ancestor. Developmental data showing different ontogeny of shells have not supported the latter opinion [103]. Diasoma has been equivocally recovered within one mitogenomic analysis ([104], but see [105] for limitations of protein coding mitochondrial genes), and in one supplementary analysis of transcriptome data [8]. Similar features such as a digging foot could be interpreted as convergent adaptations to infaunal life. 

The two aplacophoran classes Caudofoveata and Solenogastres have never been associated with either scaphopods or cephalopods in morphological studies. In our analyses aplacophorans are usually paraphyletic, but some permutations, in particular when excluding (the faster-evolving, but stringently masked) COI and 16S markers, recover a clade Aplacophora sister to Cephalopoda. Aplacophora as a clade is not rejected by AU analyses of the combined 5-marker set either (Table 3). A single origin of vermiform body plans in the cephalopod stem lineage could arguably be more parsimonious than arising twice independently. Monophyly of Aplacophora is indicated by all recent studies using multiple nuclear protein coding genes and phylogenomic data sets ([5, 7, 8]; Figure 1(b)) but not neuroanatomy [106].

Aplacophorans may share an inferred tendency of modifying the ancestral foot, they have an elongated body with a foot (or head) shield with strong retractor muscle in caudofoveates, and the atrial cavity especially in Solenogastres could be interpreted as a modified preoral hood, as remnants of a hypothesised variopod body plan. Yet there is no morphological indication for a specifically aplacophoran-cephalopod clade. Interpretation of the vermiform molluscan morphology as progenetically derived rather than reflecting a basal molluscan condition (also assumed under the Aculifera concept) actually allows for hypotheses that resolve them at any position in the molluscan tree (or makes their position impossible to recover using currently available anatomical data). Assuming that aplacophorans (once or twice independently) initially evolved into interstitial secondary worms could be correlated with precerebral ganglia present in caudofoveates [106]; these transformations have evolved many times independently in interstitial worm-like gastropod groups, which are likely progenetic [47]. Calcareous spicules also evolved many times convergently in different interstitial shell-less gastropod lineages [47] and a protective dorsal cuticle covering the body evolved within progenetic corambid sea slugs [67, 68]. “Regressive” [*sensu* [107]] traits in aplacophorans such as miniaturisation, losses of shell, tentacles, and cephalisation have been attributed to progenesis [64]. The serial dorsoventral muscle grid of aplacophorans resembles early ontogenetic stages observed in other molluscs [108] and could be paedomorphic, but it is still an adaptive innovation for nonlarval stages. The narrow bipartite radulae of aplacophorans are specialised tools for microcarnivory but also resemble some stem molluscan radula types [56]; evidence from Cambrian fossils is more congruent with an ancestral unipartite radula [109].

Our topology places aplacophorans in an unconvential position; however, there is consensus among all recent molecular studies that aplacophorans represent derived rather than plesiomorphic members of Mollusca (Figure 1). These notes on the specific feature of aplacophorans therefore are of general interest to resolving the pattern and tempo of molluscan evolution, regardless of differences between our new topology and other studies.

### 3.9. Molluscan Ancestors

The origin of molluscs is a long-standing question, and speculations on the “hypothetical ancestral mollusk” depend on character-polarity and even topological assumptions [1, 59]. Broad genomic analyses (e.g., [14–16, 22]) recovered molluscs as an early-derived offshoot of Lophotrochozoa (Spiralia), as had been proposed on morphological grounds [110]. Modern morphological studies suggest entoprocts as sister to molluscs [1], a view supported by mitochondrial genomics [105]. MicroRNA data [111] suggest Annelida is the sister to Mollusca, as recovered (but never robustly supported) by most of our analyses with a large outgroup taxon set (Suppl. Figure 1A). Our analyses did not resolve a consistent sister group to Mollusca. Yet permutations and pruning of our outgroup sampling did not affect ingroup topologies. We regard the molluscan sister group as an unanswered question, but not necessarily problematic to the question of internal molluscan phylogeny (if ingroup taxon sampling is sufficiently dense). 

Our initial morphological character mapping (Figure 1(c)) suggests that the last common ancestor of living molluscs (“LAM”) was a single-shelled conchiferan with a complex body, single (or few) paired shell retractors, single paired gills in a circumpedal mantle cavity, and an elaborated (cephalised) anterior body portion. There is little reason to assume that this hypothetical LAM resembled a chiton or monoplacophoran (e.g., [25]) or to suspect a segmented body organisation (e.g., [112]). Instead, the LAM may have resembled an untorted gastropod with a cap-like shell, perhaps similar to *Latouchella*, as assumed by morphologists before the discovery of the supposedly “living fossil” *Neopilina *and still advocated by some palaeontologists [70]. 

Our assessment of potential morphological apomorphies (Figure 1(c)) and the molecular clock results (Figure 3) would suggest that the Vendian (555 Ma) *Kimberella* [78] is a candidate stem-group mollusc appearing before the evolution of a dorsal shell field. The interpretation of *Kimberella* is controversial [113], but the true stem molluscs probably did have a large, bilaterally symmetrical body with subapical mouth on a snout with a likely bipartite radula [114], a broad ventral foot, many dorsoventral muscle bundles, and a dorsal mantle covered with a resistant dorsal cuticle with mineralised spicules, which are all molluscan features, but lacking a shell [115, 116]. During the latest Precambrian rise of predators and successive development of sediment bottoms [25], molluscan larvae or early juveniles may have calcified their plesiomorphic cap-shaped mantle cuticle for protective reasons. Answering Yochelson [117], the mollusc made a shell, but then the shell made the molluscs.

## 4. Conclusions

Only one (if any) of the dozens of proposed hypotheses on molluscan phylogeny reflects the true tree. Both the traditional palaeontological concept, with monoplacophorans giving rise to all other molluscan lineages, and the widely accepted morphocladistic Testaria hypothesis, with progressive evolution from vermiform molluscs to chitons and conchiferans [62, 118], are not supported by molecular evidence and are apparently incompatible with the chronological appearance of reliable fossils representing major molluscan lineages. 

The Aculifera concept has been supported by phylogenomic results [2, 119], whose dichotomy is not inherently contradicted by the available fossil record if the last common molluscan ancestor was small and complex and had a shell (i.e., was conchiferan rather than chiton-like). Yet the branching patterns of living clades in available phylogenomic topologies appear to be incongruent with stratigraphic evidence. The debate on molluscan phylogeny can only be progressed using all available evidence, integrating morphological, fossil, and molecular data. To provide meaningful insights, molecular approaches must include all eight molluscan classes and cover the well-known diversity of living taxa. 

Our results, despite using traditional markers that cover arguably less data than next-generation approaches, are based on a comprehensive taxon set with data quality checked exhaustively at all levels. Topologies recovered still may suffer from poor sampling especially of aplacophoran lineages and from heterogeneous evolution of ingroup clades such as cephalopods or patellogastropods. The data available, while extensive and of high quality, are small in comparison to the total genetic diversity of the phylum under study. 

Nevertheless, our data sets, regimes, and analyses support and refine the Serialia hypothesis [9]. The topological results inferred herein cannot be refuted by recent research on shell building gene expression and mollusc palaeontology. In many well-studied molluscan taxa, shells are reduced or duplicated, bodies adapted to different environments and life styles such as benthic, interstitial, or pelagic realms, and features such as mantle cavities and radulae repeatedly were transformed, often drastically and rapidly. Heterochronic processes could already have occurred in the Palaeozoic, which would be consistent with the disparity known in living molluscs but which could also obscure deeper phylogenetic signal in morphological analyses. Ultimately, such complex diversification could have led to the fossil and extant molluscs that stand apart from other (noninsect) animals in terms of species diversity, body disparity, and variation of life traits. The true reconstruction of the early radiation of molluscs still is one of the major unresolved issues in evolutionary biology. Independent molecular evidence, such as microRNAs or phylogenomic data on a similarly comprehensive and dense taxon sampling as used herein, will be needed to further test these hypotheses. 

## Supplementary Material

Figure S1. Maximum Likelihood tree of 142-taxon set; A. Tree with collapsed Gastropoda; B. Expanded gastropod clade.
Figure S2. Maximum Likelihood tree of total gastropod taxon set.
Table S1. Primer sequences and PCR conditions.
Table S2. Taxa and markers used in the initial total analyses (158-taxon data set).
Table S3. Sequences that were not considered in the analyses. A. Ambiguous BLAST results. B. Bivalve taxa with 2nd amplicon of 28S showing aberrant features.
Table S4. Models of evolution used for main analyses.
Table S5. Data and taxon partitions used in different iterations of the core analyses, including gene regions, total dataset length (bp) and gaps.
Table S6. Substitution saturation tested with DAMBE.
Table S7. Fossil calibration points and settings for prior distribution of calibrated nodes.
Table S8. Time estimates of calibration points and major molluscan groups.

## Figures and Tables

**Table 1:** Preanalyses comparing different taxon sampling and masking strategies; Mafft [30] and RNAsalsa [18] are alignment methods; Aliscore [17, 31] and Gblocks [35] are masking methods.

Dataset	Alignment treatment	Alignment length (bp)	Major changes in tree topology, compared to main topology (Figure 2, Supplementary Figure 1)
Total set (158 taxa)	Mafft-cut and paste inconsistent blocks in 18S and 28S fragments-Aliscore	10318	Annelida *s.l.* sister to Mollusca; Aplacophora monophyletic (Caudofoveata sister to Solenogastres); Patellogastropoda clusters with Cephalopoda
Total set (158 taxa)	Mafft-RNAsalsa-Aliscore	7597	Mollusca non-monophyletic; Caudofoveata, Solenogastres, Cephalopoda, and Scaphopoda cluster with Annelida *s.l.*; Neritimorpha basal sister to remaining Gastropoda; Patellogastropoda sister to partial Vetigastropoda (Lepetelloida + Vetigastropoda *s.s.*)
Total set (158 taxa)	Mafft-RNAsalsa-Gblocks	4083	Nemertea + Entoprocta + Cycliophora is sister to Mollusca; Heterobranchia is sister to remaining Mollusca; Patellogastropoda clusters with Solenogastres and Cephalopoda
Large set (142 taxa, excluding Patellogastropoda)	Mafft-Gblocks	5550	Annelida *s.l.* + Entoprocta + Cycliophora is sister to Mollusca
Large set (142 taxa, excluding Patellogastropoda)	Mafft-Aliscore	8721	Main analyses (Figure 2, Supplementary Figure 1)

**Table 2:** Sensitivity tests of individual calibration nodes used for relaxed molecular clock time estimates of major molluscan groups. Table shows influence of single calibration points on node ages of all other calibration points.

		Excluded calibration point									
		None	Diversification of Mollusca	Split Serialia/Bivalvia	Origin of Cephalopoda	Split Polyplacophora/Monoplacophora	Origin of Pteriomorpha	Origin of Caenogastropoda	Diversification of Scaphopoda	Split *Astarte/Cardita *	Diversification of Polyplacophora
Calibrated nodes	Diversification of Mollusca	551.02	683.50*	550.58	549.76	551.52	551.55	551.59	551.68	552.10	551.72
	Split Serialia/Bivalvia	530.93	533.98	523.58*	530.80	530.88	530.36	530.82	531.01	530.82	531.01
	Origin of Cephalopoda	504.92	511.40	504.40	431.05*	504.26	504.85	504.75	503.96	504.16	504.35
	Split Polyplacophora/Monoplacophora	493.06	493.44	491.79	493.68	431.68*	493.47	493.13	493.47	493.79	493.14
	Origin of Pteriomorpha	475.06	474.81	474.20	474.43	474.97	376.65*	474.56	475.20	474.16	474.82
	Origin of Caenogastropoda	421.49	422.82	421.77	421.91	422.61	421.54	326.50*	421.95	420.85	421.61
	Diversification of Scaphopoda	359.95	360.02	359.97	359.55	360.12	359.59	359.37	382.50*	359.19	359.60
	Split *Astarte/Cardita *	325.43	325.40	325.20	324.98	325.45	324.55	325.37	325.09	44.34*	325.63
	Diversification of Polyplacophora	233.44	233.49	233.37	233.18	233.75	233.42	233.63	233.57	232.71	243.67*

Bold ages marked with an asterisk (∗) indicate time estimations without calibration of this node.

**Table 3:** Testing alternative topologies against various data sets. Results of Approximately Unbiased (AU) tests with Treefinder [36], various schemes. *P*-values of AU Test executed on selected taxon and data sets. Tested tree topologies were constrained in RAxML [28]. Only meaningful tests have been executed. *P*-value > 0.05: constrained topology is not rejected; *P*-value < 0.05: constrained topology is rejected significantly; *P*-value = 0: constrained topology is rejected with high significance.

Constrained topology	142-taxon set. all markers	81-taxon set. all markers	142-taxon set. 18S + 28S + H3	Aplacophora removed from 142-taxon set. all markers
Sinusoida	0.4244	Not tested	0.2652	0.0383
Mollusca + Kamptozoa	0.0	Not tested	0.0	0.0
Mollusca + Annelida	0.7421	0.7097	0.4090	0.3876
Testaria	0.0	0.0	0.0	Not tested
Aculifera	0.0	0.0	0.0	Not tested
Aplacophora	0.6908	0.3651	0.7730	Not tested
Conchifera	0.0	0.0	0.0	0.0333
Pleistomollusca	0.6665	0.0	0.0863	0.1927
Monoplacophora + Cephalopoda	0.1389	0.0632	0.0	0.2779
Scaphopoda + Gastropoda + Bivalvia	0.0154	0.0	0.0	0.1065
Scaphopoda + Cephalopoda	0.1913	0.0	0.2527	0.6914
Scaphopoda + Cephalopoda + Gastropoda	0.0	0.0	0.0	0.7232
Scaphopoda + Gastropoda	0.8850	0.9452	0.0573	0.8271
Diasoma (Scaphopoda + Bivalvia)	0.0	0.0	0.0	0.0
Monophyletic Protobranchia	0.0219	0.0	0.1085	0.0188
Dorsoconcha	0.6830	0.1097	0.3503	0.4048
Variopoda	0.3170	0.8903	0.6497	0.5952
