# Supplementary material for: The Continuing Debate on Deep Molluscan Phylogeny: Evidence for Serialia (Mollusca, Monoplacophora + Polyplacophora)
Source: Biomed Res Int. 2013 Nov 21;2013:407072. doi: 10.1155/2013/407072 (PMC3856133; doi:10.1155/2013/407072)
Supplement: Supplementary file 1 — Figure S1. Maximum Likelihood tree of 142-taxon set; A. Tree with collapsed Gastropoda; B. Expanded gastropod clade. Figure S2. Maximum Likelihood tree of total gastropod taxon set. Table S1. Primer sequences and PCR conditions. Table S2. Taxa and markers used in the initial total analyses (158-taxon data set). Table S3. Sequences that were not considered in the analyses. A. Ambiguous BLAST results. B. Bivalve taxa with 2nd amplicon of 28S showing aberrant features. Table S4. Models of evolution used for main analyses. Table S5. Data and taxon partitions used in different iterations of the core analyses, including gene regions, total dataset length (bp) and gaps. Table S6. Substitution saturation tested with DAMBE. Table S7. Fossil calibration points and settings for prior distribution of calibrated nodes. Table S8. Time estimates of calibration points and major molluscan groups. [file 407072.f1.pdf]

Supplementary Figure 1A

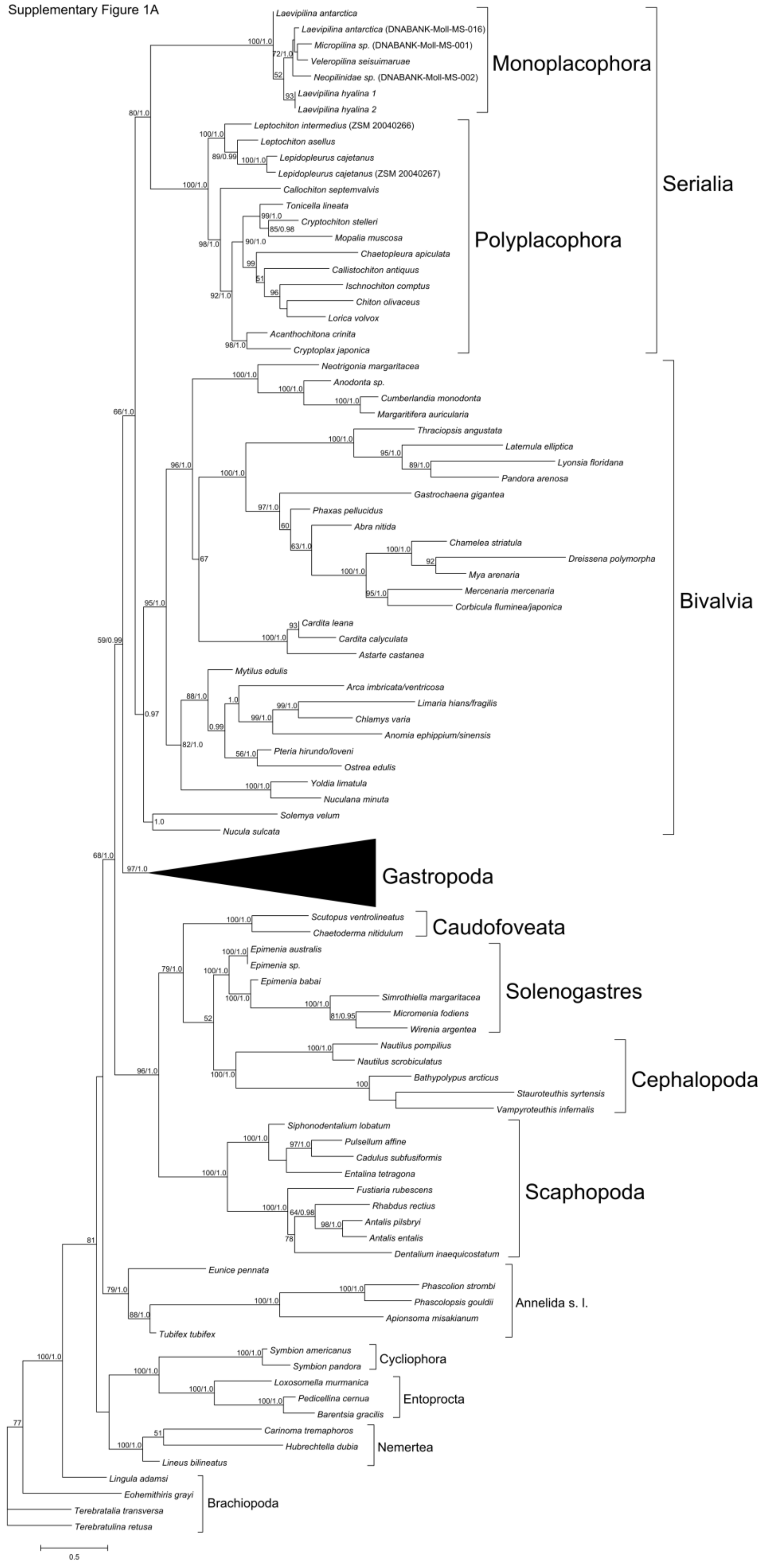

Supplementary Figure 1B

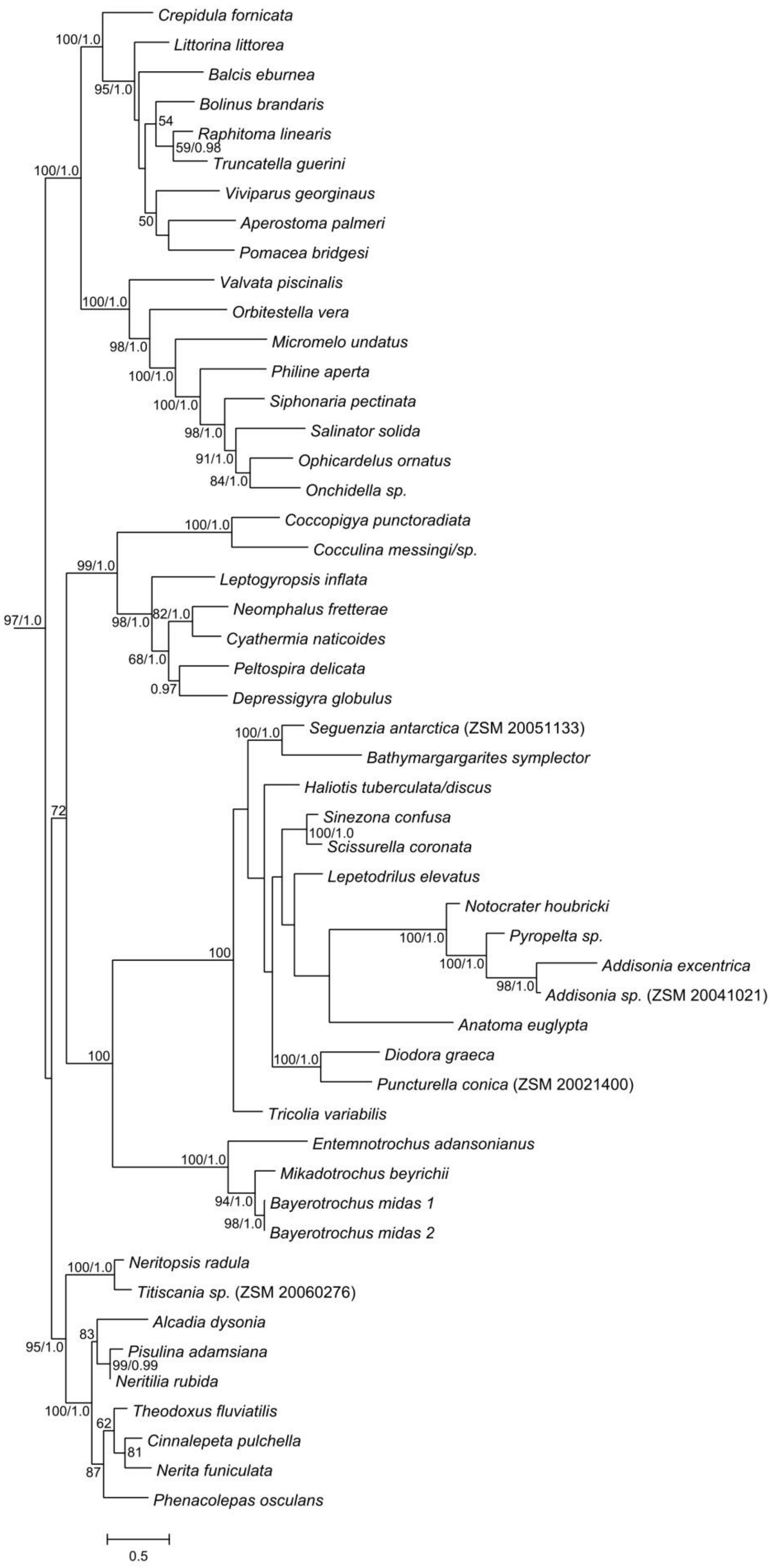

Supplementary Figure 2

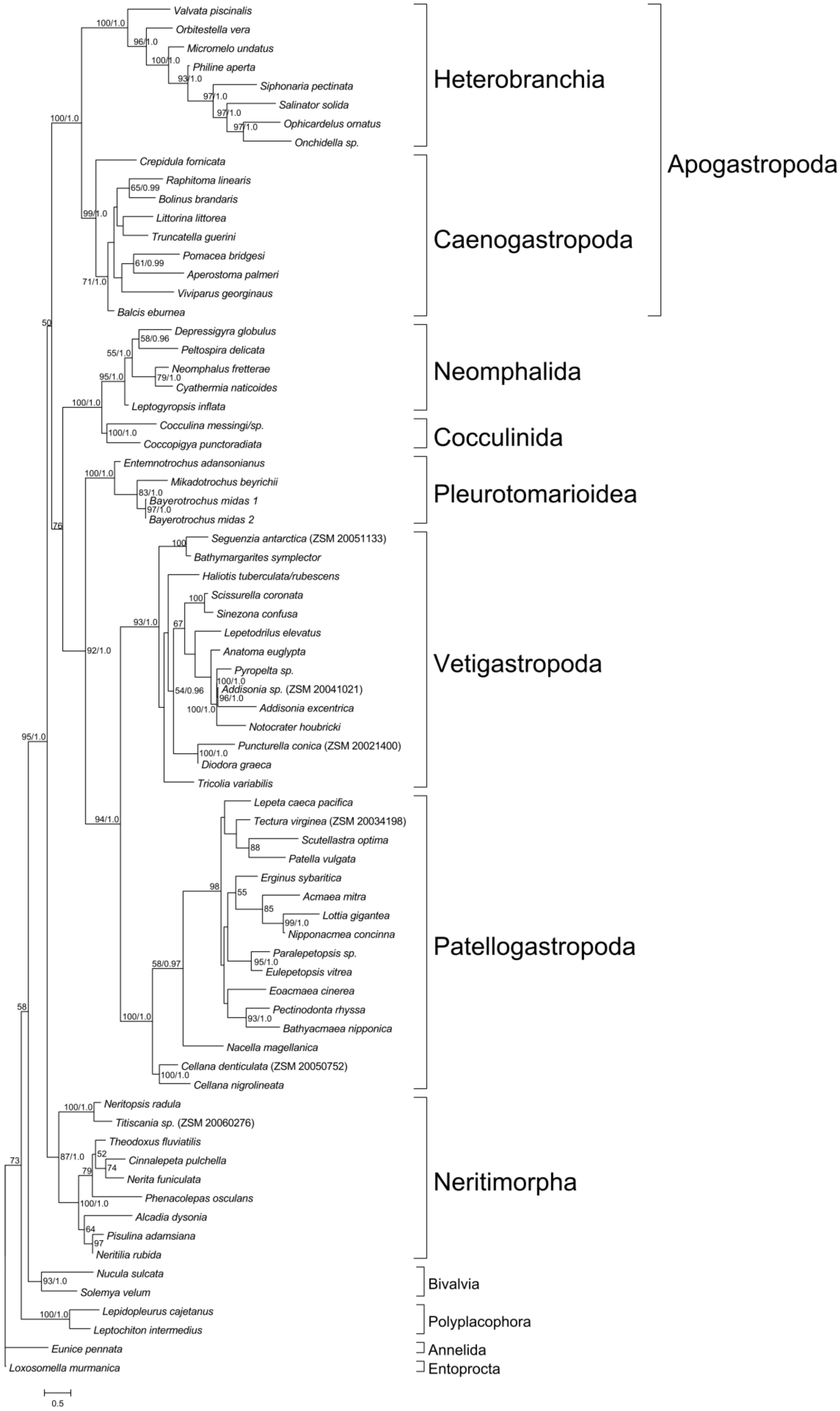

Supplementary Table 1

Fragment	Primer	Primer sequence
28S rRNA small	28SF	5'-GAC CCG TCT TGA AGC ACG-3'
	28SR	5'-CCA CAG CGC CAG TTC TGC TTA C-3'
28S rRNA large	28SF2	5'-ACC TAT TCT CAA ACT TTA AAT GG-3'
	28SR2	5'-GAC TTC CCT TAC CTA CAT-3'
18S rRNA part A	18Sa2.0	5'-ATG GTT GCA AAG CTG AAA C-3'
	18S9R	5'-GAT CCT TCC GCA GGT TCA CCT AC-3'
18S rRNA part B	18S1F	5'-TAC CTG GTT GAT CCT GCC AGT AG-3'
	18S5R	5'-CTT GGC AAA TGC TTT CGC-3'
18S rRNA part C	18S3F	5'-GTT CGA TTC CGG AGA GGG A-3'
	18Sbi	5'-GAG TCT CGT TCG TTA TCG GA-3'
16S rRNA	16Sa	5'-CGC CTG TTT ATC AAA AAC AT-3'
	16Sb	5'-CTC CGG TTT GAA CTC AGA TCA-3'
H3	H3aF	5'-ATG GCT CGT ACC AAG CAG ACV GC-3'
	H3aR	5'-ATA TCC TTR GGC ATR ATR GTG AC-3'

PCR conditions

Initial step	94°C	6min
Denaturation	94°C	1min
Annealing	50°C	1min
Elongation	72°C	1min 30sec
Final elongation	72°C	6min
Cycles		45

Supplementary Table 2

	Species	18S	28S1	28S2	28S3	28S4	H3	COI	16S
**Outgroups**									
Annelida	*Eunice pennata*	AY040684	AY340391				DQ779731		AF321418
	Tubifex tubifex	GQ355437	GQ355465	GQ355465				EF179544	EU117545
Brachiopoda	*Eohemithiris grayi*	AF025936	AY839242	AY839242	AY839242				
	Lingula adamsi	U08329							
	Terebratalia transversa	FJ196115	AF342802	AF342802	AF342802	AF342802		FJ196085	
	Terebratulina retusa	U08324	AY839244	AY839244	AY839244		DQ779768		AF334238
Cycliophora	*Symbion americanus*	EF142068		EF142087	EF142087			EF140778	EF140771
	Symbion pandora	Y14811		AY218133				AY218084	
Entoprocta	*Barentsia gracilis*	FJ196109	AY210456	AY210456	AY210456	AY210456		FJ196079	
	Loxosomella murmanica	AY218100	DQ279950	DQ279950	DQ279950	DQ279950	AY218150		
	Pedicellina cernua	FJ196111						FJ196081	
Nemertea	*Carinoma tremaphoros*	AY039675	AJ436888	AJ436888			AJ436986	AJ436943	AJ436833
	Hubrechtella dubia	AY039674	AJ436889	AJ436889				EU489495	AJ436834
	Lineus bilineatus		DQ279947	DQ279947			DQ279996	DQ280014	DQ280022
Sipuncula	*Apionsoma misakianum*	AY210440	AY210454	AY210454	AY210454	AY210454	DQ300052	EU267000	
	Phascolion strombi	DQ299984	AY210468	AY210468	AY210468	AY210468	DQ279998		
	Phascolopsis gouldii	AF342796	AF342795	AF342795	AF342795	AF342795	AF519297	DQ300134	
**Mollusca**									
Bivalvia	*Abra nitida*	DQ279940	DQ279965		DQ279965	DQ279965	DQ280005		
	Anodonta sp.	AY579090	DQ279964				AY579132	AY579122	
	Anomia ephippium/sinensis	AF120535	AB102739		AB102739	AB102739			
	Arca imbricata/ventricosa	AY654986	AB101612		AB101612	AB101612	AY654989	AY654988	
	Astarte castanea	AF120551	AF131001				DQ280004	AF120662	
	Cardita calyculata	AF120549		AF120610				AF120660	
	Cardita leana	AM774481	AM779655						
	Chamelea striatula	DQ279943		DQ279967	DQ279967	DQ279967	DQ280009	AF120668	DQ280041

	Chlamys varia	DQ279939	DQ279962		DQ279962	DQ279962	DQ280003		DQ280033
	Corbicula fluminea/japonica	AF120557	AB126330		AB126330	AB126330	AY070161	AF120666	DQ280039
	Cumberlandia monodonta	AY579105					AY579144	AY579131	AY579089
	Dreissena polymorpha	AF120552	AF131006				AY070165	AF120663	DQ280038
	Gastrochaena gigantea	AM774515	AM779689						
	Laternula elliptica	AY192687	EU734752						GU227003
	Limaria hians/fragilis	AF120534	AB102742		AB102742	AB102742	AY070152	AF120650	
	Lyonsia floridana	AF120540						AF120654	
	Margaritifera auricularia	AY579097		AY579113			AY579137	AY579125	DQ280035
	Mercenaria mercenaria	AF120559	AF131019				DQ280008	DQ399403	DQ280040
	Mya arenaria	AF120560	AB126332		AB126332	AB126332	AY377770	AY070140	AY377618
	Mytilus galloprovincialis	L33452	AB103129		AB103129	AB103129	AY267748	AY497292	AY497292
	Neotrigonia margaritacea	AF411690	DQ279963		DQ279963		AY070155	U56850	DQ280034
	Nucula sulcata	DQ279937	DQ279960		DQ279960	DQ279960	DQ280001	DQ280017	DQ280029
	Nuculana minuta	DQ279938	DQ279961		DQ279961	DQ279961	DQ280002	DQ280018	DQ280030
	Ostrea edulis	L49052	AF137047/AF120596				AY070151	AF120651	DQ280032
	Pandora arenosa	AF120539		AF120601					
	Phaxas pellucidus	DQ279941	AY145420		AY145420	AY145420	DQ280006	DQ280019	DQ280036
	Pteria hirundo/loveni	AF120532	AB102767		AB102767	AB102767		AF120647	DQ280031
	Solemya velum	AF120524	AY145421		AY145421	AY145421	AY070146	U56852	DQ280028
	Thraciopsis angustata	AM774491	AM779664						
	Yoldia limatula	AF120528	AY145424		AY145424	AY145424	AY070149	AF120642	
Caudofoveata	*Chaetoderma nitidulum*	AY377658			FJ445775		AY377763	AY377726	AY377612
	Scutopus ventrolineatus	X91977							
Cephalopoda	*Bathypolypus arcticus*	AY557465		AY557554				AF000029	DQ280044
	Nautilus pompilius	AY557455		AY145417				AY557514	AY377628
	Nautilus scrobiculatus	AF120504		AF120567			AF033704		U11606
	Stauroteuthis syrtensis	AY557457					AY557406	AF000067	DQ280042
	Vampyroteuthis infernalis	AY557459	AH012197	AH012197	AH012197		AY557408	AF000071	DQ280043
Gastropoda	*Acmaea mitra*	AB282760	AB282781	AB282781	AB282781			AB238459	AB106518

Addisonia excentrica	AY603096							
Addisonia sp. (ZSM 20041021)	KF527260		KF527269	KF527269		KF527283		
Alcadia dysonia	DQ093428		DQ279974	DQ279974		DQ093496		DQ093469
Anatoma euglypta	AY923897					AY923971	AY923934	
Aperostoma palmeri	DQ093435	DQ279983	DQ279983	DQ279983		DQ093505	DQ093523	DQ093479
Balcis eburnea	AF120519		AF120576				AF120636	DQ280051
Bathyacmaea nipponica	AB282772	AB282793					AB238588	AB238451
Bathymargarites symplector	DQ093433	DQ279982	DQ279982	DQ279982	DQ279982	DQ093503	DQ093521	DQ093477
Bayerotrochus midas 1	AF120510		DQ093453			DQ093500	AY296820	DQ093474
Bayerotrochus midas 2	FJ977637	FJ977668	FJ977668	FJ977668				
Bolinus brandaris	DQ279944	DQ279986	DQ279986	DQ279986		DQ280010	DQ280020	DQ280052
Cellana nigrolineata	DQ013353		DQ279971			DQ093493	DQ093515	DQ093467
Cinnalepeta pulchella			AB087192	AB087192				
Coccopigya punctoradiata	AB282774	AB282795					AB238590	AB238453
Cocculina messingi/sp.	AF120508	DQ279973	DQ279973	DQ279973		AY377777	AY377731	AY377624
Crepidula fornicata	AY377660					AY377778	AF353154	AY377625
Cyathermia naticoides	DQ093430	DQ279977	DQ279977	DQ279977		DQ093498	DQ093518	DQ093472
Depressigyra globulus	DQ093431	DQ279978	DQ279978	DQ279978	DQ279978	AF033689	DQ093519	DQ093473
Diodora graeca	AF120513	DQ279980	DQ279980	DQ279980	DQ279980	DQ093502	AF120632	DQ093476
Entemnotrochus adansonianus	AF120509	DQ279979	DQ279979	DQ279979		AY377774		AY377621
Eoacmaea conoidalis	AB282757	AB282778					AB238505	AB238375
Erginus sybaritica	AB282761	AB282782	AB282782	AB282782			AB238461	AB238350
Eulepetopsis vitrea	DQ093427	DQ279972	DQ279972			DQ093495	DQ093516	DQ093468
Haliotis tuberculata/discus	AF120511	AY145418	AY145418	AY145418	AY145418	AY070145	AY377729	AY377622
Lepeta caeca pacifica	AB282759	AB282780	AB282780	AB282780			AB238458	AB238347
Lepetodrilus elevatus	DQ093432	AY145413	AY145413	AY145413	AY145413	DQ093501	DQ093520	DQ093475
Leptogyropsis inflata	AB365313					AB365300	AB365258	
Littorina littorea	DQ093437	DQ279985	DQ279985			DQ093507	DQ093525	DQ093481
Lottia gigantea	AB282762	AB282783	AB282783	AB282783			AB238466	AB106498
Micromelo undatus	DQ093443		DQ279995			DQ093513		DQ093487

Mikadotrochus beyrichii	AM048636	AM048695	AM048695	AM048695		AM049331	
Nacella magellanica	AB282769	AB282790	AB282790		AB433689	EU870985	AB238433
Neomphalus fretterae	AY090806						
Nerita funiculata	DQ093429		DQ279976	DQ279976		DQ093517	DQ093471
Neritilia rubida			AB087190	AB087190			
Neritopsis radula			AB087186				
Nipponacmea concinna	DQ013354					AB238486	AB106511
Notocrater houbricki	L78881				AF033700	AY296822	
Onchidella sp.	DQ093441		DQ279992		DQ093511	DQ093529	DQ093485
Ophicardelus ornatus	DQ093442				DQ093512	DQ093530	DQ093486
Orbitestella vera	FJ917207	FJ917239	FJ917239		EF561623	FJ917268	FJ917250
Paralepetopsis sp.	FJ977635	FJ977665	FJ977665		FJ977728	FJ977752	FJ977699
Patella vulgata	AB282770	AB282791	AB282791	AB282791		AB238580	AB238445
Pectinodonta rhyssa	AB282773	AB282794	AB282794	AB282794		AB238589	AB238452
Peltospira delicata	AY923893				AY923967	AY923931	
Phenacolepas osculans	AY923890					AY923928	
Philine aperta	DQ093438	DQ279988	DQ279988	DQ279988	DQ093508		DQ093482
Pisulina adamsiana			AB087191	AB087191			
Pomacea bridgesi			DQ279984	DQ279984	DQ093506	DQ093524	DQ093480
Puncturella conica (ZSM 20021400)	KF527257		KF527266	KF527266	KF527278		KF527251
Pyropelta sp.	FJ977636	FJ977666	FJ977666	FJ977666	FJ977729	FJ977753	FJ977700
Raphitoma linearis	DQ279945		DQ279987		DQ280011		DQ280053
Salinator solida	DQ093440	DQ279991	DQ279991	DQ279991	DQ093510	DQ093528	DQ093484
Scissurella coronata	AM048637	AM048696	AM048696				
Scutellastra optima	AB282771	AB282792				AB238585	AB106482
Seguenzia antarctica (ZSM 20051133)	KF527261		KF527270	KF527270	KF527279		KF527253
Sinezona confusa	AF120512	DQ279981	DQ279981	DQ279981	AY377773		
Siphonaria pectinata	X91973	DQ279993	DQ279993		AY377780	AF120638	AY377627
Theodoxus fluviatilis	AF120515		DQ279975	DQ279975		AF120633	DQ093470
Titiscania sp. (ZSM 20060276)	KF527262		KF527271	KF527271	KF527280		KF527254

	Tricolia variabilis	AB365304					AB365267	AB365219	
	Truncatella guerini	AF120518		AF120575				AF120635	
	Valvata piscinalis	FJ917222	FJ917224	FJ917224	FJ917224	FJ917224		FJ917267	FJ917248
	Viviparus georginaus	AF120516		AF120574			AY377779	AF120634	AY377626
Monoplacophora	*Laevipilina antarctica*			DQ279958					
	Laevipilina antarctica (DNABANK-Moll-MS-016)	KF527265		KF527274	KF527274		KF527276		KF527256
	Laevipilina hyalina 1	FJ445774	FJ445777	FJ445777	FJ445777	FJ445777	FJ445778	FJ445781	FJ445782
	Laevipilina hyalina 2	FJ449542	FJ449541	FJ449541	FJ449541	FJ449541		FJ449540	FJ449543
	Micropilina sp. (DNABANK-Moll-MS-001)	KF527263		KF527272	KF527272		KF527277		KF527255
	Neopilinidae sp. (DNABANK-Moll-MS-002)	KF527264		KF527273	KF527273		KF527275		
	Veleropilina seisuimaruae	AB669192	AB669193	AB669193	AB669193	AB669193	AB669194	AB669195	AB669196
Polyplacophora	*Acanthochitona crinita*	AF120503	DQ279957	DQ279957	DQ279957		AY377759	AF120627	AY377609
	Callistochiton antiquus	AY377645	DQ279953	DQ279953	DQ279953		AY377749	AY377712	AY377599
	Callochiton septemvalvis	AY377632		DQ279952	DQ279952		AY377736	AY377700	
	Chaetopleura apiculata	AY377636	AY145398	AY145398	AY145398	AY145398	AY377741	AY377704	AY377590
	Chiton olivaceus	AY377651		DQ279955	DQ279955		AY377755	AY377716	AY377605
	Cryptochiton stelleri			AY377686			AY377760	AY377720	AY377610
	Cryptoplax japonica	AY377656	AY145402	AY145402	AY145402	AY145402	AY377761	FJ445780	AY377611
	Ischnochiton comptus	AY377639	AY145412	AY145412	AY145412	AY145412	AY377744	AY377709	AY377593
	Lepidopleurus cajetanus	AF120502	FJ445776	FJ445776	FJ445776		AY377735	AF120626	AY377585
	Lepidopleurus cajetanus (ZSM 20040267)	KF527259		KF527268	KF527268		KF527282		KF527252
	Leptochiton intermedius (ZSM 20040266)	KF527258		KF527267	KF527267		KF527281		
	Leptochiton asellus	AY377631	AY145414	AY145414	AY145414	AY145414	AY377734	FJ461256	AY377586
	Lorica volvox	AY377647	DQ279954	DQ279954	DQ279954		AY377751		AY377601
	Mopalia muscosa	AY377648	DQ279956	DQ279956	DQ279956		AY377752		AY377602
	Tonicella lineata	AY377635		AY377665			AY377739	AY377702	AY377588
Scaphopoda	*Antalis entalis*	DQ279936	AY145388	AY145388	AY145388	AY145388	DQ280000	DQ280016	DQ280027
	Antalis pilsbryi	AF120522		AF120579				AF120639	
	Cadulus subfusiformis	AF490603							
	Dentalium inaequicostatum	DQ279935		DQ279959	DQ279959		DQ279999	DQ280015	DQ280026

	Entalina tetragona	AF490598				
	Fustiaria rubescens	AF490597				
	Pulsellum affine	AF490600				
	Rhabdus rectius	AF120523	AF120580	AY377772	AF120640	AY377619
	Siphonodentalium lobatum	AF490601				
Solenogastres	*Epimenia sp.*	AY377657	AY377691	AY377765	AY377723	AY377615
	Epimenia australis		AY377689	AY377767	AY377722	AY377614
	Epimenia babai	AY212107	AY377690	AY377766	AY377724	AY377616
	Micromenia fodiens	FJ649601				
	Simrothiella margaritacea	FJ649600				
	Wirenia argentea	FJ649599				

Supplementary Table 3A

	Species	18S	28S1	28S2	28S3	28S4	H3	COI	16S
Annelida	*Eunice pennata*							AY838870	
	Lineus bilineatus	DQ279932			DQ279947	DQ279947			
Bivalvia	*Cardita calyculata*						AY070156		
	Cumberlandia monodonta		AF305382						
Brachiopoda	*Eohemithiris grayi*							AB053200	
	Lingula adamsi							AB128054	
	Terebratulina retusa							NC000941	
Cephalopoda	*Nautilus pompilius*		AY145417		AY145417	AY145417			
	Stauroteuthis syrtensis			DQ279968	DQ279968				
Cycliophora	*Symbion pandora*						AY218153		
Gastropoda	*Coccopigya punctoradiata*						AB365301		
	Crepidula fornicata		AY145406	AY145406	AY145406	AY145406			
	Nerita funiculata						DQ093497		
	Neritilia rubida							AB102712	
	Neritopsis radula				AB087186				
	Ophicardelus ornatus		DQ279994	DQ279994	DQ279994				
	Pomacea bridgesi	DQ093436							
	Scissurella coronata							AM049332	AM116867
	Sinezona confusa							AF120631	
Nemertea	*Hubrechtella dubia*						AJ436987		
Polyplacophora	*Mopalia muscosa*							AY377713	

Supplementary Table 3B

	Species	28S2
**Bivalvia**	*Gastrochaena gigantea*	AM779689
	Thraciopsis angustata	AM779664
	Solemya velum	AY145421
	Nucula sulcata	DQ279960
	Nuculana minuta	DQ279961
	Yoldia limatula	AY145424
	Mytilus galloprovincialis	AB103129
	Arca imbricata/ventricosa	AB101612
	Pteria hirundo/loveni	AB102767
	Ostrea edulis	AF137047 + AF120596
	Limaria hians/fragilis	AB102742
	Anomia ephippium/sinensis	AB102739
	Chlamys varia	DQ279962
	Neotrigonia margaritacea	DQ279963
	Anodonta sp.	DQ279964
	Abra nitida	DQ279965
	Phaxas pellucidus	AY145420
	Dreissena polymorpha	AF131006
	Corbicula fluminea / japonica	AB126330
	Mercenaria mercenaria	AF131019
	Mya arenaria	AB126332
	Astarte castanea	AF131001
	Cardita leana	AM779655

Supplementary Table 4

Data set	Number of taxa	Total length of alignment (aligned with Mafft, masked with Aliscore)	Model of sequence evolution (AIC)	Proportion of invariable sites	Gamma distribution shape parameter
**16S (142 taxa)**	87	519	TVM+I+G	0.1554	0.6522
**18S (142 taxa)**	133	3617	GTR+I+G	0.1739	0.5009
**28S (142 taxa)**	118	3583	GTR+I+G	0.2126	0.5255
**H3 (142 taxa) all codon positions**	94	322	TVM+I+G	0.5221	1.0204
**H3 (142 taxa) 1st codon position**	94	322	SYM+I	0.8382	equal
**H3 (142 taxa) 2nd codon position**	94	322	GTR+G	-	1.7209
**H3 (142 taxa) 3rd codon position**	94	322	GTR+I+G	0.5797	2.5094
**COI (142 taxa) all codon positions**	92	678	GTR+G	-	0.2944
**COI (142 taxa) 1st codon position**	92	678	GTR+I+G	0.1649	0.6211
**COI (142 taxa) 2nd codon position**	92	678	GTR+G	-	0.7885
**COI (142 taxa) 3rd codon position**	92	678	GTR+I+G	0.2253	0.8226
**16S (81 taxa)**	51	459	GTR+I+G	0.1784	0.7234
**18S (81 taxa)**	78	3449	GTR+I+G	0.1271	0.4028
**28S (81 taxa)**	68	3465	GTR+I+G	0.2235	0.4999
**H3 (81 taxa) all codon positions**	55	327	GTR+I+G	0.5395	1.3596
**H3 (81 taxa) 1st codon position**	55	327	SYM+I	0.8289	equal
**H3 (81 taxa) 2nd codon position**	55	327	SYM+I+G	0.0332	2.0347
**H3 (81 taxa) 3rd codon position**	55	327	GTR+I+G	0.5940	7.1635
**COI (81 taxa) all codon positions**	51	666	GTR+G	-	0.2723
**COI (81 taxa) 1st codon position**	51	666	GTR+I+G	0.2019	0.6262
**COI (81 taxa) 2nd codon position**	51	666	GTR+G	-	0.4874
**COI (81 taxa) 3rd codon position**	51	666	GTR+I+G	0.2320	0.7413
**16S (Gastropods)**	53	531	TVM+I+G	0.1968	0.9294
**18S (Gastropods)**	67	1959	TrN+I+G	0.1772	0.4059
**28S (Gastropods)**	62	2808	GTR+I+G	0.2945	0.4736

**H3 (Gastropods) all codon positions**	47	326	GTR+I+G	0.5564	1.3578
**H3 (Gastropods) 1st codon position**	47	326	SYM+I+G	0.7580	0.1501
**H3 (Gastropods) 2nd codon position**	47	326	GTR+I+G	0.0330	0.7695
**H3 (Gastropods) 3rd codon position**	47	326	GTR+I	0.6461	equal
**COI (Gastropods) all codon positions**	51	672	GTR+I+G	0.2489	0.4266
**COI (Gastropods) 1st codon position**	51	672	GTR+I+G	0.2541	0.6279
**COI (Gastropods) 2nd codon position**	51	672	GTR+G	-	0.6405
**COI (Gastropods) 3rd codon position**	51	672	GTR+I+G	0.2950	0.7110

Supplementary Table 5

Data set	Variations on data sets	Partition	Alignment length	proportion of gaps
142 taxa	-	9: 16S, 18S, 28S, COI 1st, 2nd, 3rd codon position, H3 1st, 2nd, 3rd codon position	8721	60%
81 taxa	-	9: 16S, 18S, 28S, COI-1st, -2nd, -3rd codon position, H3-1st, -2nd, -3rd codon position	8367	57%
134 taxa, without Aplacophora	exclusion of Aplacophora; exclusion of 3rd codon positions of COI and H3	5: 16S, 18S, 28S, COI, H3	8122	58%
74 taxa, without Aplacophora	exclusion of Aplacophora; exclusion of 3rd codon positions of COI and H3	5: 16S, 18S, 28S, COI, H3	7812	55%
142 taxa	COI and H3 coded as amino acids	5: 16S, 18S, 28S, COI (aa), H3 (aa)	8052	62% (16S-18S-28S); 25% (COI-H3)
142 taxa	only fragments 18S, 28S and H3 are used	5: 18S, 28S, H3-1st, -2nd, -3rd codon position	7527	61%
Gastropods (67 gastropods including patellogastropods, 6 outgroups)	only gastropod taxa are used (including Patellogastropoda)	9: 16S, 18S, 28S, COI-1st, -2nd, -3rd codon position, H3-1st, -2nd, -3rd codon position	6296	53%

Supplementary Table 6

**Data set**	**saturation**	**Iss**		**Iss c Sym (P)**
**H3 (142 taxa) all codon positions**	no	0,330	<	0,692 (0,0000)
**H3 (142 taxa) 1st and 2nd codon positions**	no	0,215	<	0,749 (0,0000)
**H3 (142 taxa) 3rd codon position**	no	0,192	<	0,949 (0,0000)
**COI (142 taxa) all codon positions**	no	0,410	<	0,697 (0,0000)
**COI (142 taxa) 1st and 2nd codon positions**	no	0,239	<	0,683 (0,0000)
**COI (142 taxa) 3rd codon position**	yes	0,918	>	0,720 (0,0000)
**H3 (81 taxa) all codon positions**	no	0,392	<	0,689 (0,0000)
**H3 (81 taxa) 1st and 2nd codon positions**	no	0,045	<	0,722 (0,0000)
**H3 (81 taxa) 3rd codon position**	no	0,814	<	1,039 (0,0001)
**COI (81 taxa) all codon positions**	no	0,418	<	0,703 (0,0000)
**COI (81 taxa) 1st and 2nd codon positions**	no	0,251	<	0,685 (0,0000)
**COI (81 taxa) 3rd codon position**	yes	0,925	>	0,704 (0,0000)
**H3 (Gastropods) all codon positions**	no	0,414	<	0,687 (0,0000)
**H3 (Gastropods) 1st and 2nd codon positions**	no	0,046	<	0,715 (0,0000)
**H3 (Gastropods) 3rd codon position**	no	0,842	<	1,009 (0,0001)
**COI (Gastropods) all codon positions**	no	0,466	<	0,706 (0,0000)
**COI (Gastropods) 1st and 2nd codon positions**	no	0,164	<	0,687 (0,0000)
**COI (Gastropods) 3rd codon position**	yes	0,929	>	0,700 (0,0000)

Supplementary Table 7

	Calibration node on preferred tree	Fossil calibration	Date range (Ma)	Reference	Prior settings in BEAST v. 1.6.1 (distribution; gamma shape, gamma scale, zero offset)
**1**	Mollusca	first shell record	~ 545	Parkhaev 2008	Gamma; 2.5, 2.0, 542.0
**2**	Serialia/Bivalvia	*Fordilla*	~ 530	Parkhaev 2008	Gamma; 3.3, 2.2, 525.0
**3**	Cephalopoda/Solenogastres	*Plectronoceras*	~ 505	Nishiguchi and Mapes 2008	Gamma; 2.4, 7.0, 495.0
**4**	Polyplacophora/Monoplacophora	*Orthriochiton*	~ 490	Vendrasco and Runnegar 2004	Gamma; 5.0, 5.0, 470.0
**5**	Origin of Pteriomorpha	Cyrtodontidae	~ 475	Pojeta 1978	Gamma; 1.9, 9.5, 465.0
**6**	Origin of Caenogastropoda	Sublitoidea	~ 418	Nützel et al. 2000	Gamma; 2.3, 9.0, 405.0
**7**	Scaphopoda	*Dentalium*	~ 353	Yochelson 1999	Gamma; 2.2, 6.7, 345.0
**8**	*Astarte/Cardita*	*Astartella concentrica*	~ 322	Hoare et al. 1989	Gamma; 2.6, 5.0, 315.0
**9**	Polyplacophora	*Leptochiton davolii*	~ 231	Laghi 2005	Gamma; 5.0, 2.7, 220.0

Parkhaev PY: **The early molluscan radiation.** In: *Phylogeny and evolution of the Mollusca*. Edited by Ponder WF, Lindberg DR. Berkeley, Univ. California Press 2008, 33-69.

Nishiguchi MK, Mapes RH: **Cephalopoda.** In: *Phylogeny and evolution of the Mollusca*. Edited by Ponder WF, Lindberg DR. Berkeley, Univ. California Press 2008, 163-199.

Vendrasco MJ, Runnegar B: **Late Cambrian and Early Ordovician stem group chitons (Mollusca: Polyplacophora) from Utah and Missouri.** *J Paleontol* 2004, **78**:675-689.

Pojeta J: **The origin and early taxonomic diversification of pelecypods.** *Phil Trans R Soc Lond B* 1978, **284**:225-246.

Nützel A, Erwin DH, Mapes RH: **Identity and phylogeny of the Late Paleozoic Sublitoidea (Gastropoda).** *J Paleontol* 2000, **74**:575-598.

Yochelson EL: **Scaphopoda.** In: *Functional morphology of the invertebrate skeleton*. Edited by Savazzi E. Chichester. John Wiley and Sons 1999, 363-377.

Hoare RD, Heaney III MJ, Mapes RH: **Bivalves (Mollusca) from the Imo Formation (Mississippian, Chesterian) of North-Central Arkansas.** *J Paeontol* 1989, **63**:582-603.

Laghi GF: **Upper triassic chitons from the Italian Dolomites.** *Lav Sci Nat* 2005, **30**:79-84.

Supplementary Table 8

	time estimation (BEAST v. 1.6.1)
**Calibrated nodes**	
Diversification of Mollusca	551,02 (542,88-559,94)
Split Serialia/Bivalvia	530,93 (525,89-536,72)
Split Cephalopoda/Solenogastres	504,92 (495,72-516,34)
Split Polyplacophora/Monoplacophora	493,06 (476,69-511,76)
Origin of Pteriomorpha	475,07 (465,17-487,24)
Origin of Caenogastropoda	421,49 (405,93-441,94)
Diversification of Scaphopoda	359,95 (345,61-379,28)
Split *Astarte/Cardita*	325,43 (315,69-337,77)
Diversification of Polyplacophora	233,44 (223,22-244,78)
**Major molluscan groups**	
Diversification of Dorsoconcha	541,69 (532,24-551,28)
Diversification of Bivalvia	495,69 (478,15-514,89)
Diversification of Gastropoda	493,14 (444,21-531,35)
Diversification of Monoplacophora	65,29 (30,36-110,8)
Diversification of Variopoda	534,2 (515,99-550,29)
Split Caudofoveata/Cephalopoda+Solenogastres	518,59 (501,75-536,16)
Diversification of Cephalopoda	364,19 (255,97-467,95)
Diversification of Solenogastres	195,61 (117,78-277,8)
Diversification of Caudofoveata	160,52 (19,5-274,97)
